# Programmable synthesis of multiply arylated cubanes through C–H metalation and arylation†

**DOI:** 10.1039/d0sc01909g

**Published:** 2020-04-24

**Authors:** Ryo Okude, Genki Mori, Akiko Yagi, Kenichiro Itami

**Affiliations:** Graduate School of Science, Nagoya University Chikusa Nagoya 464-8602 Japan yagi.akiko@d.mbox.nagoya-u.ac.jp; Central Pharmaceutical Research Institute, Japan Tobacco Inc. 1-1 Murasaki-cho, Takatsuki Osaka 569-1125 Japan; Institute of Transformative Bio-Molecules (WPI-ITbM), Nagoya University Chikusa Nagoya 464-8602 Japan

## Abstract

Cubane (C_8_H_8_), a cubic alkane, has long attracted attention owing to its unique 3D structure. In order to utilize the cubane scaffold widely in science and technology, a powerful method for synthesizing diverse cubane derivatives is required. Herein, we report the synthesis of mono-, di-, tri-, and tetra-arylated cubanes. Directed *ortho*-metalation with lithium base/alkyl zinc and subsequent palladium-catalyzed arylation enabled C–H metalation and arylation of cubane. This reaction allows the late-stage and regioselective installation of a wide range of aryl groups, realizing the first programmable synthesis of diverse multiply arylated cubanes.

## Abstract 2

Cubane has attracted attention due to its unique 3D structure. Herein, we report the programmable synthesis of multiply arylated cubanes. The developed reaction allows the late-stage and regioselective installation of aryl groups.

## Graphical abstract

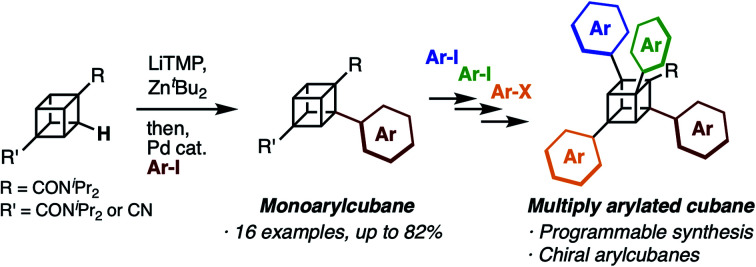

## Introduction

Structurally unique molecules have always fascinated scientists, which has driven investigations into their synthesis, functionalization, and applications. Cubane, which is a cubic alkane represented by C_8_H_8_, has particularly attracted attention over the years.^1^ The eight sp^3^-hybridized carbon atoms form C–C bonds with a small bond angle (*ca.* 90°), constructing a rigid cubic structure with high strain energy (161.5 kcal mol^−1^). Since the landmark synthesis by Eaton in 1964,^2^ the synthetic route has been greatly improved,^3^ enabling kilogram-scale synthesis to date.^4^ A number of derivatives have been created, and some of them have been found to work as high-energy materials,^5^ bioactive molecules,^6^ and ligands for metal–organic frameworks.^7^ Despite the high demand, the synthesis of cubane derivatives has been a very difficult task. Most of the reported derivatives have substituents at their 1- and 4-positions because these molecules are easily accessible from commercially available 1,4-cubanedicarboxylic acid, which is a synthetic precursor of cubane.^8^

**Fig. 1:**
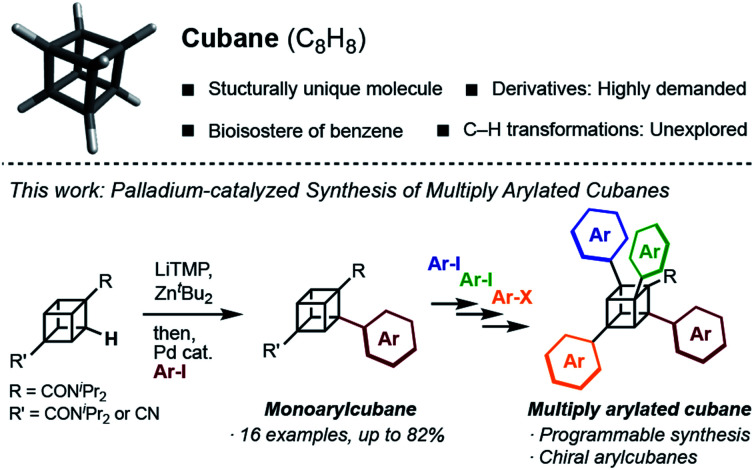
Schematic illustration of this work.

Late-stage C–H transformation of cubane is a potentially powerful strategy for rapidly generating molecular diversity in cubane derivatives, but the installable groups have mainly been limited to halogeno,^9^ carboxylic,^10^ phenyl,^11^ and hydroxy^12^ groups. The high reactivity of the C–C bonds can also cause decomposition of the cubane scaffold under various reaction conditions.^13^ To open a new vista of cubane-based molecular science, we began our studies of cubane C–H transformation chemistry, for which we selected arylation of cubane as the first and most valuable target reaction. Arylcubanes are cubane derivatives that have recently attracted attention as bioisosteres of pharmacologically important biaryls.^14^ Multiply arylated cubanes, unprecedented cubane derivatives, are also interesting for their unique, 3D, and diverse chemical spaces constructed with rigidly directed aryl groups. Herein, we report a palladium-catalyzed arylation of amidocubanes through directed *ortho*-C–H metalation. This method permits the regioselective, late-stage installation of a wide variety of aryl groups onto the cubane framework, culminating in the first synthesis of multiply arylated cubanes (Fig. 1).

In 1988, Bashir-Hashemi reported C–H phenylation of cubane, in which cubylmagnesium bromide was generated through directed *ortho*-lithiation of cubane-1,4-bis(*N*,*N*-diisopropyl-amide) (**1a**) and then treated with benzyne to give phenylcubane.^11^ In reference to this reaction, we focused on the development of arylation through directed *ortho*-metalation of **1a**. Based on the p*K*_a_ value of cubane (calculated p*K*_a_ = 40.8)^15^ and that of benzene (p*K*_a_ = 43), the metalation and arylation methods for arenes were investigated. In 1999, Kondo and Uchiyama developed a palladium-catalyzed arylation of ethyl benzoate by using lithium tetramethyl-piperidide (LiTMP) and Zn^*t*^Bu_2_.^16^ In this reaction, biaryls are most likely obtained through the generation of aryllithium zincate directed by an ester group.

## Results and discussion

**Scheme 1:**
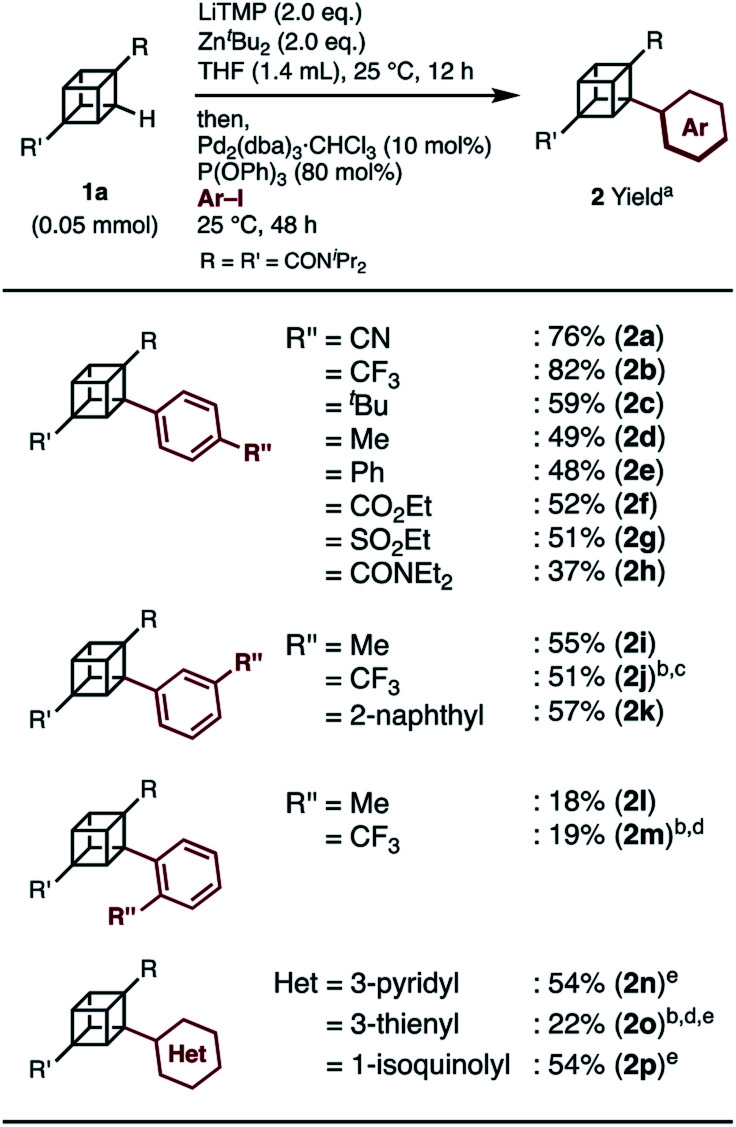
Substrate scope of iodoarenes in palladium-catalyzed arylation of cubane. ^*a*^ Isolated yield. ^*b*^ 0.05 M, Pd_2_(dba)_3_·CHCl_3_/P(OPh)_3_ = 1/4, THF solution (10 mol%), 25 °C, 24 h. ^*c*^**1a**: 0.10 mmol. ^*d*^**1a**: 0.10 mmol, *ortho*-metalation: 1 h. ^*e*^ Pd_2_(dba)_3_·CHCl_3_ (20 mol%), P(OPh)_3_ (160 mol%).

When we applied the arylation developed by Kondo and Uchiyama to **1a** and 4-iodobenzonitrile with Pd_2_(dibenzylideneacetone(dba))_3_·CHCl_3_ and PPh_3_ as catalysts, monoarylcubane **2a** was successfully obtained in 23% yield (see Table S1,† entry 1). The ligands for the palladium-catalyzed arylation were then investigated, and **2a** was obtained in 76% isolated yield by using triphenylphosphite. The regioselectivity of arylation was confirmed by X-ray single-crystal structure analysis. Parameters such as catalyst loading, reaction time, concentration, and leaving group on the arene were also investigated, leading to the conclusion that the conditions described in Scheme 1 are optimal (see Table S1† for details). Because no multiply arylated cubanes were obtained under these conditions, we presumed that a monocubyllithium zincate species was generated and reacted with an aryl palladium complex to afford the monoarylcubane. With the optimal reaction conditions in hand, the installable aryl groups were examined. Aryl iodides with electron-withdrawing groups at the *para*-position (4-CN and 4-CF_3_) gave monoarylated cubanes in excellent yields (**2a** and **2b**). Other groups, such as ester, amide, sulfonamide, Ph, ^*t*^Bu, and Me, at the *para*-position were also installed in moderate yields (**2c–2h**). Aryl iodides with *meta*-substituents reacted in acceptable yields, whereas the reactions of *ortho*-substituted arenes resulted in low yields (**2i–2m**). Heteroarenes such as pyridine, quinolone, and thiophene are also applicable in the reaction (**2n–2p**). X-ray crystal structures were successfully obtained for the monoarylcubanes **2a**, **2e**, **2f**, **2k**, and **2p** (see Fig. S1–S5†).

**Scheme 2:**
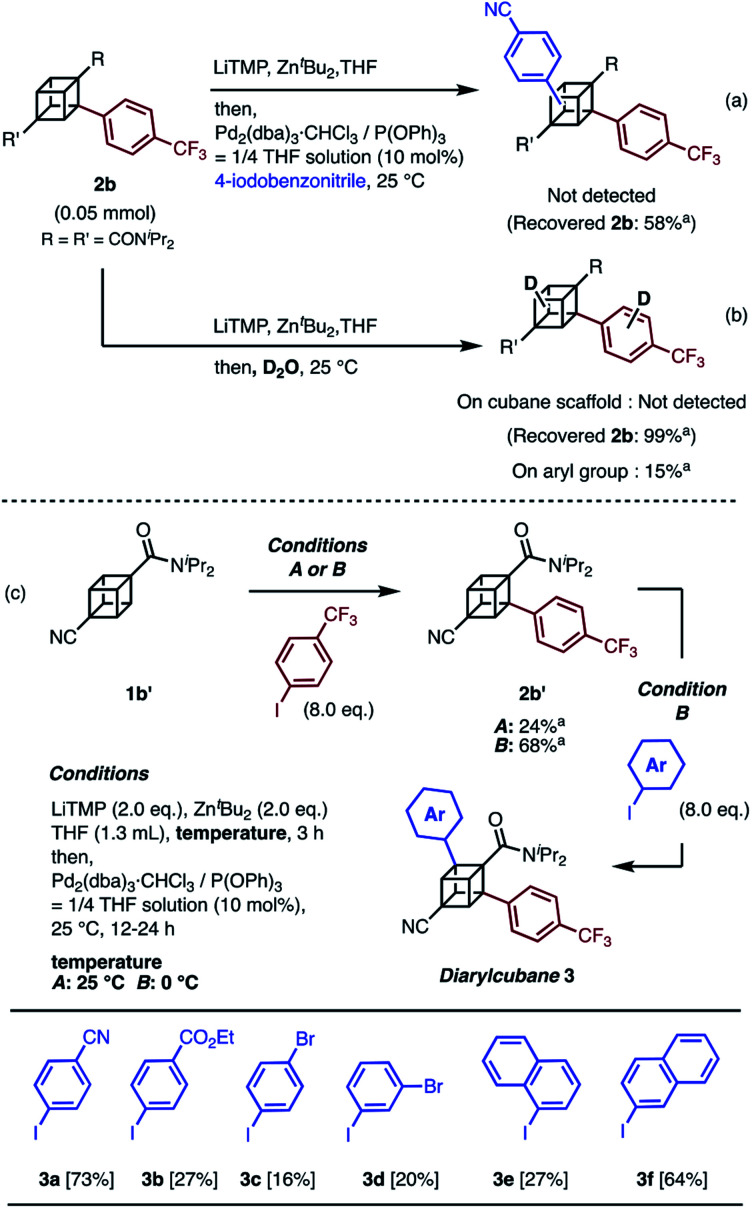
Attempts at (a) arylation of **2b** and (b) deuteration of **2b** through directed *ortho*-metalation. (c) Synthesis of diarylcubanes and scope for iodoarenes. ^*a* 1^H NMR yield.

Next, the synthesis of diarylcubanes was investigated. We expected the arylation of monoarylcubanes to give diarylcubanes. However, no further arylated product was obtained from **2b** and a 4-iodoarene (Scheme 2a). The product arylated on the trifluoromethylaryl group of **2b** was not identified, and about 40% of **2b** was decomposed. Deuteration after directed *ortho*-metalation of **2b** did not proceed on the cubane framework, indicating that the cubyllithium zincate was not generated from **2b** (Scheme 2b). In a multiple iodination reaction of a cyanocubane derivative reported by Eaton, enhanced acidity of cubane was the key for multiple functionalization.^17^ Following this principle, cyanocubane-amide **1b′** was synthesized and utilized for multiple arylation (Scheme 2c). While the reaction of **1b′** at 25 °C (Condition A) resulted in a low yield of **2b′**, the metalation of **1b′** at low temperature (−78 °C to 0 °C, Condition B) followed by arylation at 25 °C afforded **2b′** in 68% yield. Notably, **2b′** can be obtained even on a gram scale (1.1 g). The conditions used for the arylation of **1b′** also enabled the arylation of **2b′** to give diarylcubane **3a** in excellent yield (73%). The structure of **3a** was confirmed by X-ray crystal structure analysis, which proved the regioselectivity of the second aryl group (Fig. S6†). Iodoarenes with bromo and ester groups, as well as iodonaphthalenes, reacted well to give the corresponding diarylcubanes **3b–3f**. The second arylation reaction took place regioselectively at the C–H bonds near the amido group, demonstrating that the aryl-installing positions are predictable and programmable. It is notable that the diarylcubanes are chiral cubanes, which are a few cubane derivatives.^8*a*,18^ The racemic products **3a** and **3f** were separated with a chiral column to afford the chiral molecules (Fig. S9 and S10†).

**Scheme 3:**
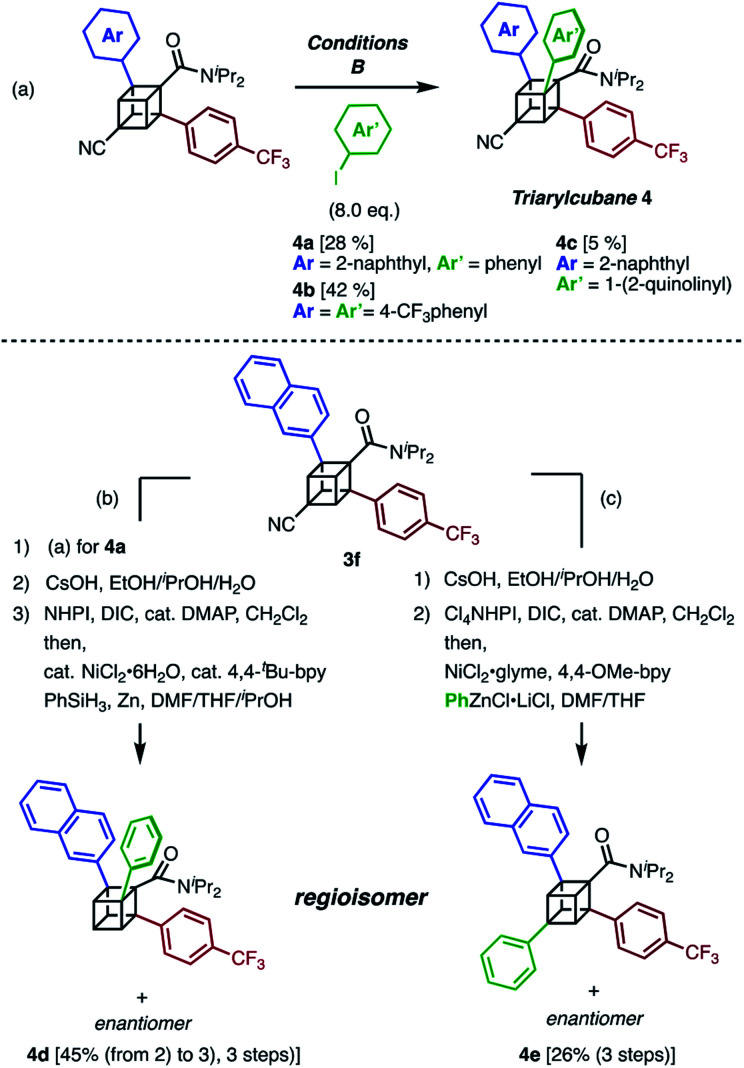
Synthesis of triarylcubanes and programmable synthesis of triarylcubane regioisomers. (a) Synthesis of triarylcubanes **4a**, **4b**, and **4c**. (b and c) Regioselective synthesis of triarylcubane isomers. (b) Arylation, hydrolysis, and decarboxylation.

The next challenge in our campaign was to achieve the first synthesis of triarylcubanes (Scheme 3a). The arylation of **3f** under our optimal conditions (Condition B) proceeded to give triarylcubanes **4a**, **4b**, and **4c**, respectively. Therefore, we envisioned that the programmable synthesis of triarylcubanes is accessible (Scheme 3b and c). Through a combination of our methods and those of Baran,^19^ the arylation and subsequent hydrolysis/esterification/decarboxylation of **3f** afforded **4d** as a racemic mixture. Triarylcubanes with three different aryl groups have several regioisomers depending on the substituent positions; one of the regioisomers of **4d** can also be accessed by transformation of the cyano group of **3f**. In accordance with a previous report,^20^ a sequence of hydrolysis, esterification, and decarboxylative arylation afforded the racemic product **4e**. The four isomers of triarylcubanes have different chemical spaces constructed by differently directed aryl groups, realizing rapid access to diverse chemical spaces through C–H arylations of cubane.

**Scheme 4:**
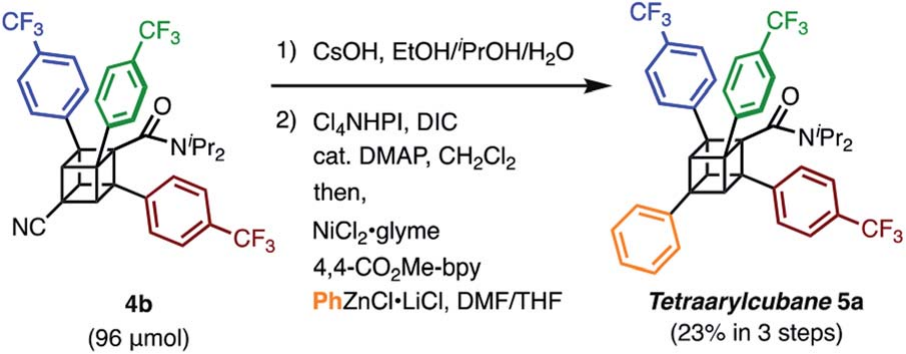
The synthesis of tetraarylcubane **5a**.

**Fig. 2:**
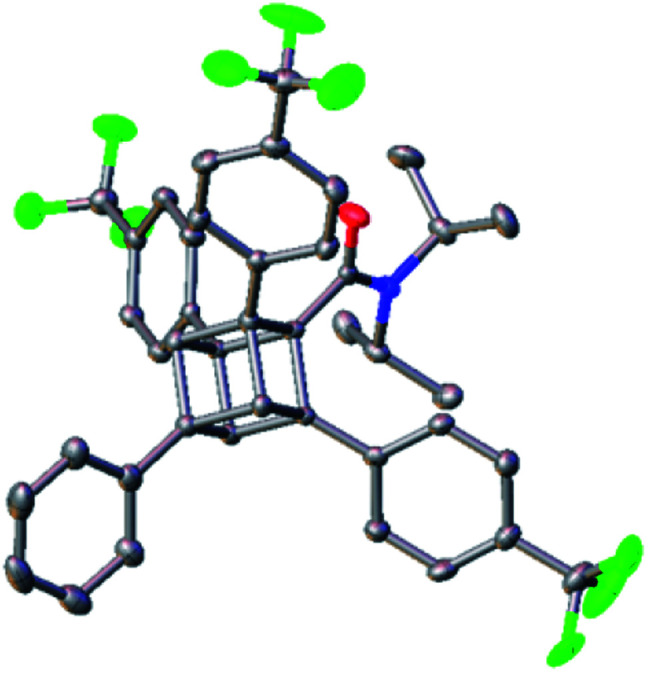
X-ray crystal structure of **5a** at 50% thermal stability.

We finally synthesized a tetraarylcubane by combining our arylation and Senge's decarboxylation. Hydrolysis and subsequent decarboxyphenylation of **4b** successfully furnished tetraarylcubane **5a** (Scheme 4), the structure of which was confirmed by X-ray crystal structure analysis (Fig. 2). The four aryl groups were introduced at the expected positions, directing four different vectors. This rigid and unique structure has potential as a novel tetrahedron unit widely used in the creation of 3D organic frameworks.

## Conclusions

In summary, we have achieved a programmable synthesis of multiply arylated cubanes through directed *ortho*-C–H metalation of amidocubanes and subsequent palladium-catalyzed cross-coupling. A wide range of aryl groups were installable regioselectively, and several kinds of mono-, di-, tri-, and tetra-arylated cubanes were synthesized. The regioselective synthesis of triarylcubane isomers is also possible, demonstrating that diverse multiply arylated cubanes are accessible by the current method. Arylcubanes are a new family of cubane derivatives, which have unique 3D chemical spaces and significant potential as functional materials and pharmaceuticals. The applications of arylcubanes in materials science and biology are now being explored in our laboratory.

## Conflicts of interest

There are no conflicts to declare.

## Supplementary Material

SC-011-D0SC01909G-s001

SC-011-D0SC01909G-s002
