# Supplementary material for: Programmable synthesis of multiply arylated cubanes through C–H metalation and arylation
Source: Chem Sci. 2020 Apr 24;11(29):7672–5. doi: 10.1039/d0sc01909g (PMC8159448; doi:10.1039/d0sc01909g)
Supplement: SC-011-D0SC01909G-s001 [file SC-011-D0SC01909G-s001.pdf]

Electronic Supplementary Material (ESI) for Chemical Science.
This journal is © The Royal Society of Chemistry 2020

Supporting Information

Programmable Synthesis of Multiply Arylated Cubanes through C–H Metalation and Arylation

Ryo Okude,[†] Genki Mori,[‡] Akiko Yagi, [†,*] and Kenichiro Itami[†,#,¶,*]

[†] Graduate School of Science, Nagoya University, Chikusa, Nagoya 464-8602, Japan.
[‡] Central Pharmaceutical Research Institute, Japan Tobacco Inc., 1-1 Murasaki-cho, Takatsuki, Osaka 569-1125, Japan.
[#] JST-ERATO, Itami Molecular Nanocarbon Project, Nagoya University, Chikusa, Nagoya 464-8602, Japan.
[¶] Institute of Transformative Bio-Molecules (WPI-ITbM), Nagoya University, Chikusa, Nagoya 464-8602, Japan.

Table of Contents

1. General S2
2. Ligand Screening S3
3. Synthesis of Starting Materials S4
4. Preparation of the Reagent $TMPZn^{t}Bu_2Li$ S4-5
5. General Procedure for Pd-catalyzed Arylation of Cubane S5-13
6. Synthesis of Multiply Arylated Cubanes S13–24
7. X-ray Crystallographic Analysis S25–31
8. HPLC Chart S32–33
9. Reference S34
10. ^{1}H and ^{13}C NMR Spectra 35–96

1. *General*

Unless otherwise noted, all reactants or reagents including dry solvents were obtained from commercial suppliers and used without further purification except for $ZnCl_2$ (vacuum drying using heat gun). $Pd_2(dba)_3$•$CHCl_3$, tris(4-(trifluoromethyl)phenyl)phosphite, *N*,*N*-diethyl-4-iodobenzenesulfonamide, *N*,*N*-diethyl-4-iodobenzenamide and 2-iodonaphthalene were prepared according to the procedures reported in the literatures.[S1-5] All reactions were performed with dry solvents under an atmosphere of N_2 gas in flame-dried glassware using standard vacuum-line techniques. Tetrahydrofuran (THF) and CH_2Cl_2 for reactions were purified by passing through a solvent purification system (Glass Contour). All work-up and purification procedures were carried out with reagent-grade solvents in air. The word "r.t. (room temperature) in this Supporting Information means 25 °C.

Analytical thin-layer chromatography (TLC) was performed using E. Merck silica gel 60 F254 precoated plates (0.25 mm). The developed chromatogram was analyzed by UV lamp (254 nm) or phosphomolybdic acid/sulfuric acid solution. Flash column chromatography was performed with E. Merck silica gel 60 (230–400 mesh). Preparative thin-layer chromatography (PTLC) was performed using Wakogel B5-F silica coated plates (0.75 mm) prepared in our laboratory. Preparative recycling gel permeation chromatography (GPC) was performed with either YMC LC-Forte/R instrument equipped with YMC-GPC T4000/YMC-GPC T30000 columns using $CHCl_3$ as an eluent, or LC-9210II NEXT instrument (Japan Analytical Industry Ltd.) equipped with an in-line JAIGEL-1H/2H or JAIGEL-1HR/2HR columns using $CHCl_3$ as an eluent. The high-resolution mass spectra (HRMS) were conducted on Thermo Fisher Scientific Exactive. Nuclear magnetic resonance (NMR) spectra were recorded on a JEOL JNM-ECA-400 (^{1}H 400 MHz, ^{13}C 100 MHz), a JEOL JNM-ECA-500 (^{1}H 500 MHz, ^{13}C 126 MHz) and JEOL JNM-ECA-600 (^{1}H 600 MHz, ^{13}C 150 MHz) spectrometer. Chemical shifts for ^{1}H NMR are expressed in parts per million (ppm) relative to tetramethylsilane (δ 0.00 ppm) or residual peak of CD_3CN (δ 1.94 ppm), acetone-d_6 (δ 2.05 ppm), CD_2Cl_2 (δ 5.32 ppm), cyclohexane-d_{12} (δ 1.38 ppm). Chemical shifts for ^{13}C NMR are expressed in ppm relative to $CDCl_3$ (δ 77.1 ppm) or cyclohexane-d_{12} (δ 26.4 ppm). Data are reported as follows: chemical shift, multiplicity (s = singlet, d = doublet, dd = doublet of doublets, ddd = doublet of doublets of doublets, t = triplet, td = triplet of doublets, q = quartet, dq = doublet of quartets, sep = septet, m = multiplet, brs = broad singlet, brd = broad doublet), coupling constant (Hz), and integration. NMR yields were determined using dibromomethane as an internal NMR standard.

2. ***Ligand Screening***

Note : Reaction procedure is same as General Procedure 1 in Section 5.

Table S1. Ligands screening for C–H arylation of cubane diamide **1a**

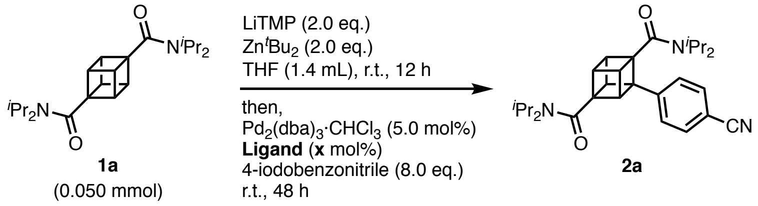

entry	**Ligand**	**x**	**2a**[a]
1	PPh_3	40	28%
2	P(2-furyl)3	40	N.D.
3	dppe	40	2%
4	dppp	40	13%
5	$P(OPh)_3$	40	58%
**6**[b]	$P(OPh)_3$	**80**	**84%**
7[b]	$P(OPh)_3$	60	19%
8[b]	$P(OPh)_3$	100	3%
9	$P(OEt)_3$	40	1%
10	$P(OC_6H_4CF_3)_3$	40	<1%

[a] ^{1}H NMR yield

[b] $Pd_2(dba)_3 \cdot CHCl_3$ (10 mol%)

3. ***Synthesis of Starting Materials***

Cubane-1,4-bis(*N*,*N*-diisopropylamide) **1a**

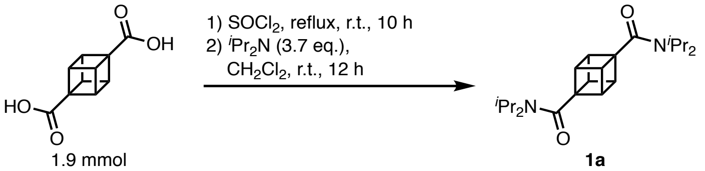

To a 100-mL two-necked round-bottomed flask containing a magnetic stirring bar were added 1,4-cubanedicarboxylic acid (365 mg, 1.9 mmol) and thionyl chloride (4.0 mL) under N_2 atmosphere. After heated at reflux for 10 h, excess thionyl chloride was removed under reduced pressure and the obtained residue was dissolved in CH_2Cl_2 (11 mL). Diisopropylamine (1.0 mL, 7.1 mmol, 3.7 equiv) were added with ice cooling and stirring. After stirring for 12 h at room temperature, the reaction was quenched with aq. Na_2CO_3 (10 mL). The amide was extracted with CH_2Cl_2 (10 mL x 3). The organic layer was dried over Na_2SO_4 (ca. 5 g) and concentrated in vacuo. The purification by column chromatography on silica gel (EtOAc only) gave **1a** as a white solid (540 mg, 1.5 mmol, 79%). ^1H NMR (600 MHz, $CDCl_3$) δ 4.14 (s, 6H), 3.51 (sep, J = 6.5 Hz, 2H), 3.31 (sep, J = 6.9 Hz, 2H), 1.42 (d, J = 6.9 Hz, 12H), 1.22 (d, J = 6.5 Hz, 12H) ppm. ^{13}C NMR (151 MHz, $CDCl_3$) δ 170.5, 58.5, 48.5, 46.3, 46.0, 21.2, 20.7 ppm. HR-MS (ESI-MS, positive): *m/z* = 359.2691 calcd for $C_{22}H_{35}N_2O_2$: 359.2693 $[M + H]^+$.

Cyanocubaneamide **1b'**

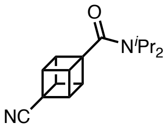

1b' was prepared from 1,4-cubanedicarboxylic acid following a literature procedure.[S6] Recrystallized from THF/*n*-hexane, white solid. Spectral and physical data in accordance with the literature.[S6]

4. ***Preparation of the Reagent TMPZn^{t}Bu$_2$Li***

Preparation of LiTMP solution [S7]

According to a literature procedure, a 20-mL Schlenk tube equipped with J. Young® O-ring tap containing a magnetic stirring bar was charged with 2,2,6,6-tetramethylpiperidine (TMP-H) (190 μL, 1.1 mmol, 1.10 equiv) and THF (2.0 mL) in a glovebox under argon. The mixture

was cooled at −78 ºC and ^{n}BuLi (1.59 M in *n*-hexane, 630 μL, 1.0 mmol, 1.0 equiv) was added to the mixture. The mixture was stirred at 0 ºC for 1 h.

Preparation of TMPZn^{t}Bu$_2$Li solution (0.17 M in THF) [S7]

According to a literature procedure, a 20-mL Schlenk tube equipped with J. Young® O-ring tap containing a magnetic stirring bar was charged with $ZnCl_2$ (136 mg, 1.0 mmol, 1.0 equiv) and THF (2.0 mL) in a glovebox under argon. The mixture was cooled at −78 ºC and ^{t}BuLi (1.61 M in *n*-pentane, 1.3 mL, 2.1 mmol, 2.1 equiv) was added to the mixture. The mixture was stirred at 0 ºC for 1 h and LiTMP solution was added to the mixture at −78 ºC. The mixture was stirred at 0 ºC for 1 h to give TMPZn^{t}Bu$_2$Li solution (0.17 M in THF).

5. *General Procedure for Pd-catalyzed Arylation of Cubane*

General Procedure 1

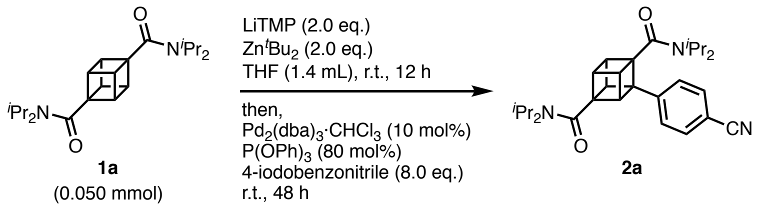

A 20-mL Schlenk tube equipped with J. Young® O-ring tap containing a magnetic stirring bar was charged with **1a** (17.9 mg, 0.050 mmol) and THF (800 μL) under N_2 atmosphere. To the mixture, TMPZn^{t}Bu$_2$Li solution (0.17 M in THF, 600 μL, 0.10 mmol, 2.0 equiv) was added at room temperature. After stirring at room temperature for 12 h, $Pd_2(dba)_3$•$CHCl_3$ (5.2 mg, 5.0 μmol, 10 mol%), triphenyl phosphite (11 μL, 40 μmol, 80 mol%) and 4-iodobenzonitrile (91.6 mg, 0.40 mmol, 8.0 equiv) were added and the solution was stirred for 48 h. Then the reaction mixture was quenched with aq. NH_4Cl. Then resulting mixture was extracted with CH_2Cl_2 three times. The organic extracts were dried over Na_2SO_4, and concentrated. The residue was then purified by silica gel chromatography (EtOAc/$CHCl_3$ = 1/10) and then GPC.

General Procedure 2

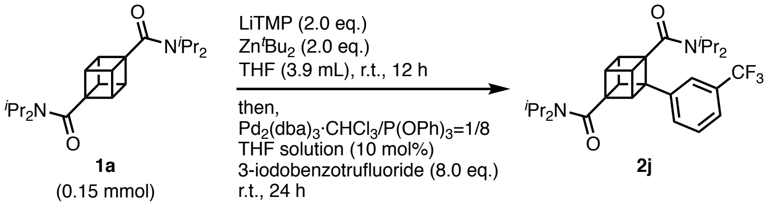

A 20-mL Schlenk tube equipped with J. Young® O-ring tap containing a magnetic stirring bar was charged with **1a** (53.8 mg, 0.15 mmol) and THF (2.1 mL) under N_2 atmosphere. To the mixture, TMPZn^{t}Bu$_2$Li solution (0.17 M in THF, 1.8 mL, 0.30 mmol, 2.0 equiv) was added at

room temperature and the reaction mixture was stirred at room temperature for 12 h. $Pd_2(dba)_3$•$CHCl_3$/$P(OPh)_3$ solution ($Pd_2(dba)_3$•$CHCl_3$:0.050 M in THF) was prepared from $Pd_2(dba)_3$•$CHCl_3$ (51.8 mg, 0.050 mmol), triphenyl phosphite (105 μL, 0.40 mmol) and THF (1.0 mL). To the reaction mixture, $Pd_2(dba)_3$•$CHCl_3$/$P(OPh)_3$ solution ($Pd_2(dba)_3$•$CHCl_3$: 0.050 M in THF, 300 μL, 15 μmol, 10 mol%) and 3-iodobenzotrifluoride (173 μL, 1.2 mmol, 8.0 equiv) were added and the solution was stirred for 24 h. Then the reaction mixture was quenched with aq. NH_4Cl. Then resulting mixture was extracted with CH_2Cl_2 three times. The organic extracts were dried over Na_2SO_4, and concentrated. The residue was then purified by silica gel chromatography (EtOAc/$CHCl_3$ = 1/10) and then GPC.

4-CN arylcubane **2a**

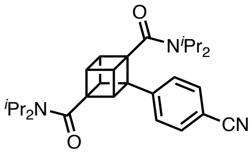

2a was prepared according to General Procedure 1 as a white solid. Purification by silica gel chromatography (EtOAc/$CHCl_3$ = 1/10) and then GPC gave **2a** (17.6 mg, 0.038 mmol, 76%). ^{1}H NMR (600 MHz, $CDCl_3$) δ 7.64 (d, J = 8.5 Hz, 2H), 7.29 (d, J = 8.6 Hz, 2H), 4.37–4.40 (m, 2H), 4.24–4.28 (m, 2H), 4.17–4.20 (m, 1H), 3.49 (sep, J = 6.6 Hz, 1H), 3.31 (sep, J = 6.8 Hz, 1H), 3.12 (sep, J = 6.8 Hz, 1H), 3.06 (sep, J = 6.5 Hz, 1H), 1.41 (d, J = 6.8 Hz, 6H), 1.30 (d, J = 6.8 Hz, 6H), 1.17 (d, J = 6.7 Hz, 6H), 0.64 (d, J = 6.5 Hz, 6H) ppm. ^{13}C NMR (150 MHz, $CDCl_3$) δ 169.5, 168.0, 144.4, 132.5, 126.5, 118.9, 110.7, 62.7, 59.4, 55.4, 50.3, 48.8, 48.8, 46.8, 46.2, 46.1, 43.6, 21.1, 20.9, 20.7, 20.5 ppm. HR-MS (ESI-MS, positive) : m/z = 482.2773. calcd for $C_{29}H_{37}N_3NaO$: 482.2778 $[M + Na]^+$.

4-CF_3 arylcubane **2b**

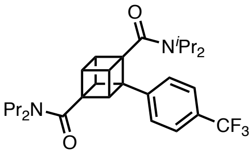

2b was prepared according to General Procedure 1 with 4-iodobenzotrifluoride (58 μL, 0.40 mmol) as a white solid. Purification by silica gel chromatography (EtOAc/$CHCl_3$ = 1/10) and then GPC gave **2b** (20.6 mg, 0.041 mmol, 82%). ^{1}H NMR (600 MHz, $CDCl_3$) δ 7.60 (d, J = 8.0 Hz, 2H), 7.29 (d, J = 8.0 Hz, 2H), 4.36–4.40 (m, 2H), 4.23–4.28 (m, 2H), 4.18–4.22 (m, 1H), 3.51 (sep, J = 6.5 Hz, 1H), 3.30 (sep, J = 6.7 Hz, 1H), 3.03–3.15 (m, 2H), 1.42 (d, J = 6.7 Hz, 6H), 1.30 (d, J = 6.7 Hz, 6H), 1.16 (d, J = 6.7 Hz, 6H), 0.61 (d, J = 6.5 Hz, 6H) ppm. ^{13}C NMR (150 MHz, $CDCl_3$) δ 169.7, 168.3, 143.1, 129.3 (q, J = 33.0 Hz), 126.0, 125.6 (d, J

= 4.3 Hz), 124.2 (q, J = 270.0 Hz), 62.6, 59.4, 55.4, 50.4, 48.8, 48.7, 46.6, 46.2, 46.1, 43.6, 21.1, 20.8, 20.7, 20.5 ppm. HR-MS (ESI-MS, positive) : m/z = 525.2699. calcd for $C_{29}H_{37}N_2NaO_2F_3$: 525.2699 $[M + Na]^+$.

4-^{t}Bu arylcubane **2c**

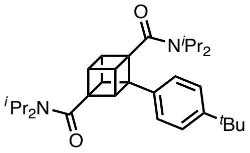

2c was prepared according to General Procedure 1 with 1-tert-butyl-4-iodobenzene (71 μL, 0.40 mmol) as a white solid. Purification by silica gel chromatography (EtOAc/$CHCl_3$ = 1/10) and then PTLC (EtOAc/benzene = 1/2) gave **2c** (14.5 mg, 0.030 mmol, 59%). ^{1}H NMR (600 MHz, $CDCl_3$) δ 7.34 (d, J = 8.6 Hz, 2H), 7.10 (d, J = 8.6 Hz, 2H), 4.33–4.36 (m, 2H), 4.20–4.23 (m, 2H), 4.16–4.20 (m, 1H), 3.57 (sep, J = 6.7 Hz, 1H), 3.29 (sep, J = 6.7 Hz, 1H), 3.04–3.12 (m, 2H), 1.41 (d, J = 6.7 Hz, 6H), 1.30 (d, J = 6.7 Hz, 6H), 1.28 (s, 9H), 1.15 (d, J = 6.5 Hz, 6H), 0.55 (d, J = 6.7 Hz, 6H) ppm. ^{13}C NMR (150 MHz, $CDCl_3$) δ 170.2, 169.0, 150.2, 136.1, 125.4, 125.3, 62.7, 59.7, 55.2, 50.3, 48.9, 48.6, 46.5, 46.1, 45.9, 43.4, 34.7, 31.5, 21.1, 20.7, 20.5, 20.5 ppm. HR-MS (ESI-MS, positive) : m/z = 513.3449. calcd for $C_{32}H_{46}N_2NaO_2$: 513.3451 $[M + Na]^+$.

4-Me arylcubane **2d**

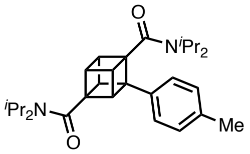

2d was prepared according to General Procedure 1 with 4-iodotoluene (87.2 mg, 0.40 mmol) as a white solid. Purification by silica gel chromatography (EtOAc/$CHCl_3$ = 1/10) and then GPC gave **2d** (11.0 mg, 0.025 mmol, 49%). ^{1}H NMR (600 MHz, $CDCl_3$) δ 7.13 (d, J = 7.9 Hz, 2H), 7.05 (d, J = 8.0 Hz, 2H), 4.30–4.33 (m, 2H), 4.17–4.23 (m, 3H), 3.54 (sep, J = 6.6 Hz, 1H), 3.28 (sep, J = 6.8 Hz, 1H), 3.05–3.16 (m, 2H), 2.32 (s, 3H), 1.41 (d, J = 6.8 Hz, 6H), 1.31 (d, J = 6.8 Hz, 6H), 1.13 (d, J = 6.6 Hz, 6H), 0.60 (d, J = 6.5 Hz, 6H) ppm. ^{13}C NMR (151 MHz, $CDCl_3$) δ 170.2, 169.0, 136.6, 136.2, 129.3, 125.5, 62.6, 59.8, 55.2, 50.5, 48.9, 48.6, 46.3, 46.1, 45.9, 43.3, 21.3, 21.0, 20.7, 20.7, 20.5 ppm. HR-MS (ESI-MS, positive) : m/z = 471.2976. calcd for $C_{29}H_{40}N_2NaO_2$: 471.2982 $[M + Na]^+$.

4-Ph arylcubane **2e**

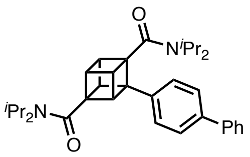

2e was prepared according to General Procedure 1 with 4-iodobiphenyl (112.0 mg, 0.40 mmol) as a white solid. Purification by silica gel chromatography (EtOAc/$CHCl_3$ = 1/10) and then GPC gave **2e** (12.2 mg, 0.024 mmol, 48%). ^{1}H NMR (600 MHz, acetone-d_6) δ 7.70 (d, J = 8.2 Hz, 2H), 7.66 (dd, J = 8.4, 1.2 Hz, 2H), 7.45 (t, J = 7.7 Hz, 2H), 7.35 (t, J = 7.4 Hz, 1H), 7.30 (d, J = 8.2 Hz, 2H), 4.42–4.45 (m, 2H), 4.21–4.25 (m, 2H), 4.15–4.18 (m, 1H), 3.65 (sep, J = 6.9 Hz, 1H), 3.39 (sep, J = 6.9 Hz, 1H), 3.25 (sep, J = 6.9 Hz, 1H), 3.18 (sep, J = 6.9 Hz, 1H), 1.38 (d, J = 6.9 Hz, 6H), 1.28 (d, J = 6.9 Hz, 6H), 1.20 (d, J = 6.9 Hz, 6H), 0.68 (d, J = 6.5 Hz, 6H) ppm. ^{13}C NMR (151 MHz, $CDCl_3$) δ 170.1, 168.8, 140.8, 139.8, 138.2, 129.0, 127.5, 127.3, 127.1, 126.1, 62.7, 59.7, 55.3, 50.5, 48.9, 48.7, 46.4, 46.1, 46.0, 43.5, 21.1, 20.8, 20.7, 20.6 ppm. HR-MS (ESI-MS, positive) : m/z = 533.3138 calcd for $C_{34}H_{42}N_2NaO_2$: 533.3138 $[M + Na]^+$.

4-CO2Et arylcubane **2f**

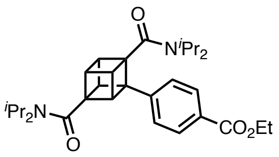

2f was prepared according to General Procedure 1 with ethyl 4-iodobenzoate (67 µL, 0.40 mmol) as a pale-orange solid. Purification by silica gel chromatography (EtOAc/$CHCl_3$ = 1/10) and then GPC gave **2f** (13.1 mg, 0.026 mmol, 52%). ^{1}H NMR (600 MHz, $CDCl_3$) δ 8.02 (d, J = 8.3 Hz, 2H), 7.24 (d, J = 8.3 Hz, 2H), 4.35–4.40 (m, 4H), 4.23–4.27 (m, 2H), 4.19–4.23 (m, 1H), 3.50 (sep, J = 6.6 Hz, 1H), 3.30 (sep, J = 6.8 Hz, 1H), 3.05–3.13 (m, 2H), 1.38–1.44 (m, 9H), 1.30 (d, J = 6.8 Hz, 6H), 1.14 (d, J = 6.6 Hz, 6H), 0.61 (d, J = 6.6 Hz, 6H) ppm. ^{13}C NMR (151 MHz, $CDCl_3$) δ 169.8, 168.4, 166.5, 144.2, 130.0, 129.1, 125.5, 62.7, 61.2, 59.7, 55.4, 50.6, 48.8, 48.7, 46.4, 46.2, 46.0, 43.5, 21.0, 20.9, 20.7, 20.5, 14.5 ppm. HR-MS (ESI-MS, positive) : m/z = 529.3037 calcd for $C_{31}H_{42}N_2NaO_4$: 529.3037 $[M + Na]^+$.

4-SO2NEt2 arylcubane **2g**

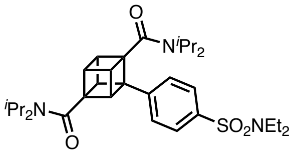

2g was prepared according to General Procedure 1 with *N*,*N*-diethyl-4-iodobenzene sulfonamide (135.7 mg, 0.40 mmol) as a white solid. Purification by silica gel chromatography (EtOAc/$CHCl_3$ = 1/10) and then PTLC (CH_3CN/CH_2Cl_2 = 1/3) gave **2g** (14.6 mg, 0.026 mmol, 51%). ^{1}H NMR (600 MHz, $CDCl_3$) δ 7.79 (d, *J* = 8.6 Hz, 2H), 7.30 (d, *J* = 8.6 Hz, 2H), 4.37–4.40 (m, 2H), 4.24–4.28 (m, 2H), 4.17–4.21 (m, 1H), 3.50 (sep, *J* = 6.7 Hz, 1H), 3.31 (sep, *J* = 6.8 Hz, 1H), 3.20 (q, *J* = 7.2 Hz, 4H), 3.03–3.14 (m, 2H), 1.42 (d, *J* = 6.7 Hz, 6H), 1.29 (d, *J* = 6.7 Hz, 6H), 1.16 (d, *J* = 6.6 Hz, 6H), 1.12 (t, *J* = 7.1 Hz, 6H), 0.62 (d, *J* = 6.5 Hz, 6H) ppm. ^{13}C NMR (151 MHz, $CDCl_3$) δ 169.7, 168.1, 143.7, 139.0, 127.5, 126.2, 62.7, 59.4, 55.4, 50.3, 48.8, 48.7, 46.7, 46.2, 46.1, 43.6, 42.1, 21.1, 20.9, 20.7, 20.5, 14.3 ppm. HR-MS (ESI-MS, positive) : *m/z* = 592.3177. calcd for $C_{32}H_{47}N_3NaO_4S$: 592.3179 $[M + Na]^+$.

4-CONEt2 arylcubane **2h**

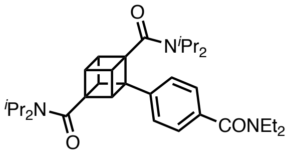

2h was prepared according to General Procedure 1 with *N*,*N*-diethyl-4-iodobenzenamide (121.3 mg, 0.40 mmol) as a white solid. Purification by silica gel chromatography (EtOAc/$CHCl_3$ = 1/10) and then PTLC (THF/benzene = 1/1) gave **2h** (9.9 mg, 0.019 mmol, 37%). ^{1}H NMR (600 MHz, $CDCl_3$) δ 7.35 (d, *J* = 8.2 Hz, 2H), 7.20 (d, *J* = 8.3 Hz, 2H), 4.34–4.37 (m, 2H), 4.21–4.25 (m, 2H), 4.18–4.21 (m, 1H), 3.46–3.57 (m, 3H), 3.29 (sep, *J* = 6.8 Hz, 1H), 3.14–3.25 (brs, 2H), 3.05–3.14 (m, 2H), 1.41 (d, *J* = 6.7 Hz, 6H), 1.29 (d, *J* = 6.8 Hz, 6H), 1.18–1.27 (brs, 3H), 1.13 (d, *J* = 6.6 Hz, 6H), 0.99–1.11 (brs, 3H), 0.62 (d, *J* = 6.5 Hz, 6H) ppm. ^{13}C NMR (151 MHz, $CDCl_3$) δ 171.1, 170.0, 168.6, 140.2, 136.0, 126.8, 125. 6, 62.6, 59.6, 55.3, 50.5, 48.7, 48.7, 46.3, 46.2, 46.0, 43.5, 43.4, 39.6, 21.0, 20.8, 20.7, 20.5, 14.3, 13.1 ppm. HR-MS (ESI-MS, positive) : *m/z* = 556.3505. calcd for $C_{33}H_{47}N_3NaO_3$: 556.3510 $[M + Na]^+$.

3-Me arylcubane **2i**

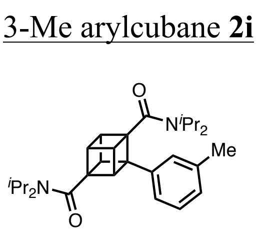

2i was prepared according to General Procedure 1 with 3-iodotoluene (51 μL, 0.40 mmol) as a white solid. Purification by silica gel chromatography (EtOAc/$CHCl_3$ = 1/10) and then PTLC (EtOAc/benzene = 1/2) gave **2i** (12.3 mg, 0.027 mmol, 55%). ^{1}H NMR (600 MHz,

$CDCl_3$) δ 7.22 (t, *J* = 7.6 Hz, 1H), δ 7.02 (d, *J* = 7.6 Hz, 1H), δ 6.98 (s, 1H), δ 6.96 (d, *J* = 7.6 Hz, 1H), 4.33–4.35 (m, 2H), 4.17–4.24 (m, 3H), 3.55 (sep, *J* = 6.6 Hz, 1H), 3.29 (sep, *J* = 6.8 Hz, 1H), 3.04–3.15 (m, 2H), 2.31 (s, 3H), 1.41 (d, *J* = 6.8 Hz, 6H), 1.31 (d, *J* = 6.8 Hz, 6H), 1.14 (d, *J* = 6.6 Hz, 6H), 0.58 (d, *J* = 6.5 Hz, 6H) ppm. ^{13}C NMR (151 MHz, $CDCl_3$) δ 170.2, 168.9, 139.0, 138.3, 128.5, 127.7, 126.2, 122.5, 62.6, 59.9, 55.2, 50.4, 48.9, 48.6, 46.3, 46.1, 45.9, 43.4, 21.4, 21.0, 20.7, 20.7, 20.5 ppm. HR-MS (ESI-MS, positive) : *m/z* = 471.2978 calcd for $C_{29}H_{40}N_2NaO_2$: 471.2982 $[M + Na]^+$.

3-CF_3 arylcubane **2j**

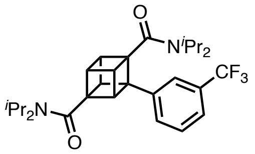

2j was prepared according to General Procedure 2 as a white solid. Purification by silica gel chromatography (EtOAc/$CHCl_3$ = 1/10) and then GPC gave **2j** (38.5 mg, 0.077 mmol, 51%). ^{1}H NMR (600 MHz, $CDCl_3$) δ 7.45–7.52 (m, 2H), 7.37–7.42 (m, 2H), 4.38–4.41 (m, 2H), 4.24–4.28 (m, 2H), 4.19–4.23 (m, 1H), 3.51 (sep, *J* = 6.7 Hz, 1H), 3.30 (sep, *J* = 6.7 Hz, 1H), 3.03–3.13 (m, 2H), 1.42 (d, *J* = 6.7 Hz, 6H), 1.29 (d, *J* = 6.7 Hz, 6H), 1.15 (d, *J* = 6.7 Hz, 6H), 0.60 (d, *J* = 6.7 Hz, 6H) ppm. ^{13}C NMR (151 MHz, $CDCl_3$) δ 169.8, 168.2, 140.2, 131.2 (q, *J* = 31.8 Hz), 129.3, 129.2, 124.1 (q, *J* = 273.2 Hz), 123.8 (d, *J* = 4.5 Hz), 122.2 (d, *J* = 3.0 Hz), 62.6, 59.4, 55.3, 50.3, 48.9, 48.7, 46.4, 46.1, 46.0, 43.5, 21.0, 20.7, 20.7, 20.4 ppm. HR-MS (ESI-MS, positive) : *m/z* = 525.2692 calcd for $C_{29}H_{37}N_2NaO_2F_3$: 525.2699 $[M + Na]^+$.

2-naphthyl arylcubane **2k**

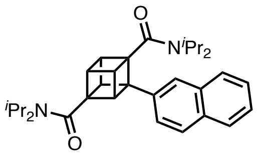

2k was prepared according to General Procedure 1 with 2-iodonaphthalene (59.8 mg, 0.40 mmol) as a white solid. Purification by silica gel chromatography (EtOAc/$CHCl_3$ = 1/10) and then GPC gave **2k** (13.9 mg, 0.029 mmol, 57%). ^{1}H NMR (500 MHz, $CDCl_3$) δ 7.76–7.84 (m, 3H), 7.59 (s, 1H), 7.43–7.50 (m, 2H), 7.31 (dd, *J* = 8.6, 1.7 Hz, 1H), 4.43–4.48 (m, 2H), 4.26–4.31 (m, 2H), 4.22–4.26 (m, 1H), 3.60 (sep, *J* = 6.6 Hz, 1H), 3.30 (sep, *J* = 6.9 Hz, 1H), 3.19 (sep, *J* = 6.6 Hz, 1H), 3.03 (sep, *J* = 6.9 Hz, 1H), 1.42 (d, *J* = 6.9 Hz, 6H), 1.29 (d, *J* = 6.9 Hz, 6H), 1.14 (d, *J* = 6.6 Hz, 6H), 0.49 (d, *J* = 6.6 Hz, 6H) ppm. ^{13}C NMR (126 MHz, $CDCl_3$) δ 170.2, 168.9, 136.7, 133.4, 132.5, 128.6, 127.9, 127.8, 126.5, 125.9, 123.9, 123.8, 62.7, 60.2,

55.3, 50.5, 48.9, 48.7, 46.4, 46.1, 45.9, 43.5, 21.0, 20.8, 20.7, 20.5 ppm. HR-MS (ESI-MS, positive) : *m/z* = 507.2978. calcd for $C_{32}H_{40}N_2NaO_2$: 507.2982 $[M + Na]^+$.

2-Me arylcubane **2l**

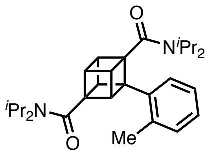

2l was prepared according to General Procedure 1 with 2-iodotoluene (51 μL, 0.40 mmol) as a white solid. Purification by silica gel chromatography (EtOAc/$CHCl_3$ = 1/10) and then PTLC (EtOAc/benzene = 1/2) gave **2l** (4.0 mg, 8.9 μmol, 18%). ^{1}H NMR (600 MHz, $CDCl_3$) δ 7.08–7.19 (m, 4H), 4.44–4.49 (m, 2H), 4.21–4.27 (m, 2H), 4.17–4.21 (m, 1H), 3.51 (sep, *J* = 6.5 Hz, 1H), 3.28 (sep, *J* = 6.7 Hz, 1H), 3.06–3.18 (m, 2H), 2.22 (s, 3H), 1.40 (d, *J* = 6.7 Hz, 6H), 1.30 (d, *J* = 6.9 Hz, 6H), 1.11 (d, *J* = 6.5 Hz, 6H), 0.57 (d, *J* = 6.4 Hz, 6H) ppm. ^{13}C NMR (151 MHz, $CDCl_3$) δ 170.2, 169.8, 137.5, 136.5, 130.8, 127.4, 126.5, 125.7, 63.1, 62.3, 54.7, 49.1, 48.6, 46.1, 45.9, 42.7, 21.0, 20.8, 20.7, 20.4, 19.6 ppm. HR-MS (ESI-MS, positive) : *m/z* = 471.2977 calcd for $C_{29}H_{40}N_2NaO_2$: 471.2982 $[M + Na]^+$.

2-CF_3 arylcubane **2m**

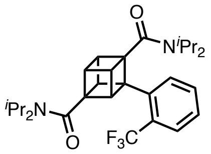

2m was prepared according to General Procedure 2 with **1a** (35.9 mg, 0.10 mmol) and 2-iodobenzotrifluoride (112 μL, 0.80 mmol) as a white solid (the reaction time with **1a** and TMPZn^{t}Bu$_2$Li : 1 h). Purification by silica gel chromatography (EtOAc/$CHCl_3$ = 1/10) and then PTLC (EtOAc/benzene = 1/2) gave **2m** (9.5 mg, 18 μmol, 19%). ^{1}H NMR (600 MHz, $CDCl_3$) δ 7.66 (d, *J* = 7.7 Hz, 1H), 7.51–7.56 (m, 1H), 7.33–7.38 (m, 2H), 4.49–4.53 (m, 2H), 4.18–4.23 (m, 2H), 4.10–4.14 (m, 1H), 3.48 (sep, *J* = 6.5 Hz, 1H), 3.29 (sep, *J* = 6.7 Hz, 1H), 3.20 (sep, *J* = 6.5 Hz, 1H), 3.13 (sep, *J* = 6.7 Hz, 1H), 1.40 (d, *J* = 6.7 Hz, 6H), 1.31 (d, *J* = 6.9 Hz, 6H), 1.14 (d, *J* = 6.7 Hz, 6H), 0.68 (d, *J* = 6.5 Hz, 6H) ppm. ^{13}C NMR (151 MHz, $CDCl_3$) δ 169.9, 168.7, 137.0, 131.9, 128.8, 127.8 (d, *J* = 31.7 Hz), 127.4 (q, *J* = 6.0 Hz), 127.3, 124.4 (q, *J* = 273.3 Hz), 64.0, 60.8, 54.7, 50.9, 48.7, 48.6, 46.5, 46.1, 46.0, 43.3, 21.0, 20.9, 20.7, 20.4 ppm. HR-MS (ESI-MS, positive) : *m/z* = 525.2701 calcd for $C_{29}H_{37}N_2NaO_2F_3$: 525.2699 $[M + Na]^+$.

3-pyridyl arylcubane **2n**

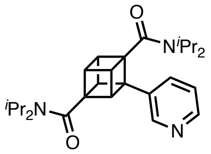

2n was prepared according to General Procedure 1 with $Pd_2(dba)_3$•$CHCl_3$ (10.4 mg, 10 μmol, 20 mol%), triphenyl phosphite (21 μL, 80 μmol, 160 mol%) and 3-iodopyridine (82.0 mg, 0.40 mmol) as a white solid. Purification by silica gel chromatography (EtOAc/$CHCl_3$ = 1/10) and then PTLC (MeOH/benzene = 1/3) gave **2n** (11.7 mg, 0.027 mmol, 54%). ^{1}H NMR (600 MHz, CD_2Cl_2) δ 8.45 (dd, *J* = 4.8, 1.4 Hz, 1H), 8.41 (d, *J* = 1.7 Hz, 1H), 7.48 (ddd, *J* = 7.9, 2.4, 1.7 Hz, 1H), 7.27 (ddd, *J* = 7.9, 4.8, 0.7 Hz, 1H), 4.37–4.41 (m, 2H), 4.23 (t, *J* = 5.0 Hz, 2H), 4.12–4.16 (m, 1H), 3.51 (sep, *J* = 6.5 Hz, 1H), 3.29 (sep, *J* = 6.9 Hz, 1H), 3.06–3.18 (m, 2H), 1.37 (d, *J* = 6.9 Hz, 6H), 1.26 (d, *J* = 6.5 Hz, 6H), 1.15 (d, *J* = 6.5 Hz, 6H), 0.65 (d, *J* = 6.5 Hz, 6H) ppm. ^{13}C NMR (151 MHz, $CDCl_3$) δ 169.7, 168.2, 148.5, 147.0, 134.5, 133.5, 123.6, 62.5, 57.6, 55.5, 50.1, 49.0, 48.7, 46.5, 46.2, 46.0, 43.7, 21.1, 20.9, 20.7, 20.5 ppm. HR-MS (ESI-MS, positive) : *m/z* = 458.2772. calcd for $C_{27}H_{37}N_3NaO_2$: 458.2778 $[M + Na]^+$.

3-thienyl arylcubane **2o**

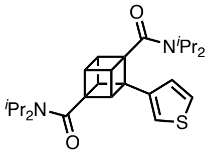

2o was prepared according to General Procedure 2 with **1a** (35.9 mg, 0.10 mmol), $Pd_2(dba)_3$•$CHCl_3$/$P(OPh)_3$ solution ($Pd_2(dba)_3$•$CHCl_3$:0.050 M in THF, 400 μL, 20 μmol, 20 mol%) and 3-iodothiophene (81 μL, 0.80 mmol) as a white solid (the reaction time for cross coupling : 48 h). Purification by silica gel chromatography (EtOAc/$CHCl_3$ = 1/10) and then PTLC (Acetone/benzene = 1/3 + one drop of NEt_3) gave **2o** (9.9 mg, 22 μmol, 22%). ^{1}H NMR (600 MHz, $CDCl_3$) δ 7.30 (dd, *J* = 5.0, 2.9 Hz, 1H), 7.00 (dd, *J* = 2.9, 1.2 Hz, 1H), 6.96 (dd, *J* = 5.0, 1.2 Hz, 1H), 4.27–4.30 (m, 2H), 4.20–4.23 (m, 2H), 4.15–4.19 (m, 1H), 3.57 (sep, *J* = 6.7 Hz, 1H), 3.30 (sep, *J* = 6.7 Hz, 1H), 3.20 (sep, *J* = 6.5 Hz, 1H), 3.14 (sep, *J* = 6.9 Hz, 1H), 1.41 (d, *J* = 6.7 Hz, 6H), 1.33 (d, *J* = 6.9 Hz, 6H), 1.15 (d, *J* = 6.5 Hz, 6H), 0.69 (d, *J* = 6.7 Hz, 6H) ppm. ^{13}C NMR (151 MHz, $CDCl_3$) δ 170.1, 168.9, 140.8, 126.7, 125.7, 120.3, 62.8, 56.6, 55.5, 51.0, 49.2, 48.6, 46.3, 46.1, 46.0, 43.6, 21.1, 20.7, 20.5 ppm. HR-MS (ESI-MS, positive) : *m/z* = 463.2379 calcd for $C_{26}H_{36}N_2NaO_2S$: 463.2390 $[M + Na]^+$.

1-isoquinolinyl arylcubane **2p**

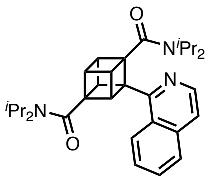

2p was prepared according to General Procedure 1 with $Pd_2(dba)_3$•$CHCl_3$ (10.4 mg, 10 μmol, 20 mol%), triphenyl phosphite (21 μL, 80 μmol, 160 mol%) and 1-iodoisoquinoline (102.0 mg, 0.40 mmol) as a white solid. Purification by silica gel chromatography (EtOAc/$CHCl_3$ = 1/10) and then PTLC (EtOAc/benzene = 1/2) gave **2p** (13.1 mg, 0.027 mmol, 54%). ^{1}H NMR (600 MHz, $CDCl_3$) δ 8.47 (d, J = 5.7 Hz, 1H), 7.85 (dd, J = 8.4, 0.7 Hz, 1H), 7.81 (d, J = 8.1 Hz, 1H), 7.65–7.69 (m, 1H), 7.58–7.62 (m, 1H), 7.51 (d, J = 5.4 Hz, 1H), 4.72–4.80 (brs, 2H), 4.40–4.47 (m, 2H), 4.32–4.36 (m, 1H), 3.58 (sep, J = 6.6 Hz, 1H), 3.28 (sep, J = 6.8 Hz, 1H), 3.22 (sep, J = 6.5 Hz, 1H), 2.86 (sep, J = 6.8 Hz, 1H), 1.41 (d, J = 6.8 Hz, 6H), 1.06 (d, J = 6.6 Hz, 6H), 0.94 (d, J = 6.6 Hz, 6H), 0.41 (d, J = 5.1 Hz, 6H) ppm. ^{13}C NMR (151 MHz, $CDCl_3$) δ 170.2, 168.9, 158.3, 141.9, 136.1, 130.5, 127.5, 127.2, 126.6, 125.8, 119.9, 63.3, 62.2, 56.1, 50.3, 49.1, 48.6, 46.1, 45.6, 45.2, 43.7, 21.0, 20.7, 20.1 ppm. HR-MS (ESI-MS, positive) : m/z = 508.2929 calcd for $C_{31}H_{39}N_3NaO_2$: 508.2934 $[M + Na]^+$.

6. ***Synthesis of Multiply Arylated Cubanes***

Synthesis of monoarylcubane **2b'**

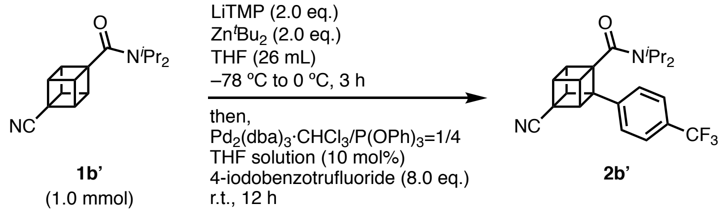

A 200-mL Schlenk tube equipped with J. Young® O-ring tap containing a magnetic stirring bar was charged with **1b'** (1.28 g, 5.0 mmol) and THF (25 mL) under N_2 atmosphere. To the mixture, TMPZn^{t}Bu$_2$Li solution (0.17 M in THF, 12 mL, 2.0 mmol, 2.0 equiv) was added at − 78 ºC and the reaction mixture was stirred at 0 ºC for 3 h. $Pd_2(dba)_3$•$CHCl_3$/$P(OPh)_3$ solution ($Pd_2(dba)_3$•$CHCl_3$:0.050 M in THF) was prepared from $Pd_2(dba)_3$•$CHCl_3$ (621 mg, 0.60 mmol), triphenyl phosphite (1.26 mL, 4.8 mmol) and THF (12 mL). To the reaction mixture, $Pd_2(dba)_3$•$CHCl_3$/$P(OPh)_3$ solution ($Pd_2(dba)_3$•$CHCl_3$:0.050 M in THF, 12 mL, 0.60 mmol, 10 mol%) was added in one portion. To this, 4-iodobenzotrifluoride (6.0 mL, 42 mmol, 8.0 equiv) was added dropwise over 10 min and the solution was stirred for 15 h at room temperature. Then the reaction mixture was quenched with aq. HCl. Then resulting mixture

was extracted with EtOAc two times. The organic extracts were washed with water, brine, dried over $MgSO_4$, and concentrated. The residue was then purified by silica gel chromatography (eluent *n*-hexane/EtOAc 10:0 to 10:3.5 with slow gradient). The first crop crystal of **2b'** (1.01 g, white crystal) was obtained by recrystallization from *n*-hexane/EtOAc. The filtrate was purified by GPC. The second crop solid of **2b'** (0.11 g) was obtained by washing with *n*-hexane. **2b'** (1.12 g, 2.8 mmol, 56%) was obtained in total. ^{1}H NMR (600 MHz, CD_3CN) δ 7.69 (d, *J* = 7.9 Hz, 2H), 7.35 (d, *J* = 7.9 Hz, 2H), 4.56–4.60 (m, 2H), 4.26–4.30 (m, 3H), 3.15 (sep, *J* = 6.9 Hz, 1H), 2.99 (sep, *J* = 6.5 Hz, 1H), 1.20 (d, *J* = 6.9 Hz, 6H), 0.61 (d, *J* = 6.5 Hz, 6H) ppm. ^{13}C NMR (151 MHz, $CDCl_3$) δ 169.7, 168.3, 143.1, 129.3 (q, *J* = 33.3 Hz), 126.0, 125.6 (d, *J* = 4.3 Hz), 124.2 (q, *J* = 271.8 Hz), 62.6, 59.4, 55.4, 50.4, 48.8, 48.7, 46.6, 46.2, 46.1, 43.6, 21.1, 20.8, 20.7, 20.5 ppm. HR-MS (ESI-MS, positive) : *m/z* = 423.1651 calcd for $C_{23}H_{23}N_2NaOF_3$: 423.1655 $[M + Na]^+$.

Synthesis of diarylcubane **3a** (General Procedure 3)

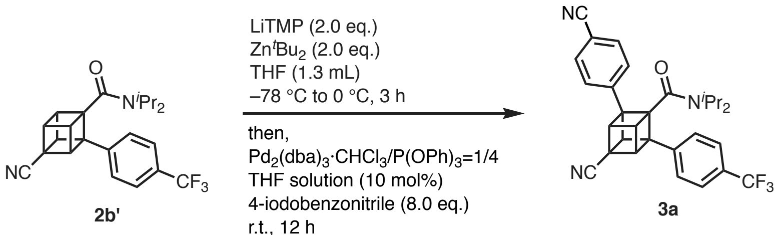

2b'
(0.050 mmol)

3a

A 20-mL Schlenk tube equipped with J. Young® O-ring tap containing a magnetic stirring bar was charged with **2b'** (20.0 mg, 0.050 mmol) and THF (700 μL) under N_2 atmosphere. To the mixture, TMPZn^{t}Bu$_2$Li solution (0.17 M in THF, 600 μL, 0.10 mmol, 2.0 equiv) was added at −78 ºC and the reaction mixture was stirred at 0 ºC for 3 h. $Pd_2(dba)_3$•$CHCl_3$/$P(OPh)_3$ solution ($Pd_2(dba)_3$•$CHCl_3$:0.050 M in THF) was prepared from $Pd_2(dba)_3$•$CHCl_3$ (51.8 mg, 0.050 mmol), triphenyl phosphite (105 μL, 0.40 mmol) and THF (1.0 mL). To the reaction mixture, $Pd_2(dba)_3$•$CHCl_3$/$P(OPh)_3$ solution ($Pd_2(dba)_3$•$CHCl_3$:0.050 M in THF, 100 μL, 5.0 μmol, 10 mol%) and 4-iodobenzonitrile (91.6 mg, 0.40 mmol, 8.0 equiv) were added and the solution was stirred for 12 h at room temperature. Then the reaction mixture was quenched with aq. NH_4Cl. Then resulting mixture was extracted with $CHCl_3$ three times. The organic extracts were dried over Na_2SO_4, and concentrated. The residue was then purified by silica gel chromatography (EtOAc/$CHCl_3$ = 1/10) and then GPC. **3a** (18.4 mg, 0.037 mmol, 73%) was obtained as a white solid. ^{1}H NMR (600 MHz, $CDCl_3$) δ 7.71 (d, *J* = 8.6 Hz, 2H), 7.67 (d, *J* = 7.9 Hz, 2H), 7.33–7.36 (m, 4H), 5.02–5.05 (m, 1H), 4.61 (td, *J* = 5.5, 0.7 Hz, 1H), 4.44–4.48 (m, 2H), 2.99 (sep, *J* = 6.5 Hz, 1H), 2.68 (sep, *J* = 6.5 Hz, 1H), 1.17–1.22 (m, 6H), 0.12 (d, *J* = 6.2 Hz, 3H), 0.07 (d, *J* = 6.2 Hz, 3H) ppm. ^{13}C NMR (151 MHz, $CDCl_3$) δ 165.0, 142.5,

141.1, 132.8, 130.3 (q, J = 33.3 Hz), 126.7, 126.1 (d, J = 5.8 Hz), 124.0 (q, J = 273.2 Hz), 118.5, 117.7, 111.7, 67.9, 59.2, 59.0, 52.8, 52.6, 51.4, 49.2, 46.2, 42.5, 34.1, 20.6, 20.5, 20.3 ppm. HR-MS (ESI-MS, positive) : m/z = 524.1919 calcd for $C_{30}H_{26}N_3NaOF_3$: 524.1920 $[M + Na]^+$.

Diarylcubane **3b**

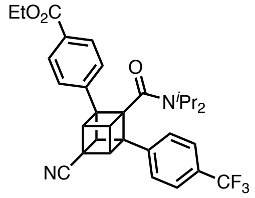

3b was prepared according to General Procedure 3 with **2b'** (60.1 mg, 0.15 mmol) and ethyl 4-iodobenzoate (200 μL, 1.2 mmol) as a pale orange solid (the reaction time for cross coupling : 18 h). Purification by silica gel chromatography (EtOAc/$CHCl_3$ = 1/10) and then GPC. **3b** (22.3 mg, 0.041 mmol, 27%) was obtained by recrystallization from *n*-hexane. ^{1}H NMR (600 MHz, CD_2Cl_2) δ 8.06 (d, J = 8.2 Hz, 2H), 7.68 (d, J = 8.2 Hz, 2H), 7.39 (d, J = 8.2 Hz, 2H), 7.32 (d, J = 8.6 Hz, 2H), 4.98–5.00 (m, 1H), 4.48–4.54 (m, 3H), 4.35 (q, J = 7.1 Hz, 2H), 2.96 (sep, J = 6.9 Hz, 1H), 2.74 (sep, J = 6.5 Hz, 1H), 1.38 (t, J = 7.2 Hz, 3H), 1.12–1.19 (m, 6H), 0.18 (d, J = 6.2 Hz, 3H), 0.12 (d, J = 6.5 Hz, 3H) ppm. ^{13}C NMR (151 MHz, $CDCl_3$) δ 166.2, 165.3, 142.1, 141.4, 130.4, 130.2 (q, J = 33.3 Hz), 130.0, 126.2, 126.1 (d, J = 2.9 Hz), 125.7, 124.0 (q, J = 273.2 Hz), 118.0, 68.0, 61.4, 59.5, 59.2, 53.2, 50.6, 49.4, 46.1, 41.9, 34.0, 20.6, 20.3, 20.3, 14.5 ppm. HR-MS (ESI-MS, positive) : m/z = 549.2357 calcd for $C_{32}H_{32}N_2O_3F_3$: 549.2360 $[M + H]^+$.

Diarylcubane **3c**

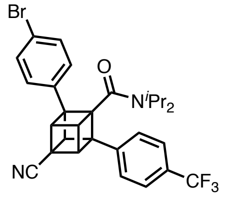

3c was prepared according to General Procedure 3 with **2b'** (80.1 mg, 0.20 mmol) and 1-bromo-4-iodobenzene (452.7 mg, 1.6 mmol) as a white solid (the reaction time for cross coupling : 24 h). Purification by silica gel chromatography (EtOAc/$CHCl_3$ = 1/10) and then GPC. **3c** (17.8 mg, 0.032 mmol, 16%) was obtained by recrystallization from *n*-hexane/THF. ^{1}H NMR (600 MHz, $CDCl_3$) δ 7.66 (d, J = 7.9 Hz, 2H), 7.54 (d, J = 8.6 Hz, 2H), 7.34 (d, J = 8.2 Hz, 2H), 7.10 (d, J = 8.2 Hz, 2H), 5.04–5.06 (m, 1H), 4.61 (t, J = 5.5 Hz, 1H), 4.35–4.39 (m, 2H), 2.96 (sep, J = 6.5 Hz, 1H), 2.65 (sep, J = 6.5 Hz, 1H), 1.19–1.24 (m, 6H), 0.07 (d, J = 6.5 Hz, 3H), −0.03 (d, J = 6.5 Hz, 3H) ppm. ^{13}C NMR (151 MHz, $CDCl_3$) δ 165.4, 141.5,

136.4, 132.3, 130.1 (q, J = 31.8 Hz), 127.5, 126.2, 126.1 (d, J = 2.9 Hz), 124.0 (q, J = 273.3 Hz), 121.9, 118.0, 67.8, 59.3, 59.1, 53.1, 53.0, 50.6, 49.4, 46.1, 41.9, 33.9, 20.6, 20.4, 20.3 ppm. HR-MS (ESI-MS, positive) : m/z = 555.1257 calcd for $C_{29}H_{27}N_2OBrF_3$: 555.1253 $[M + H]^+$.

Diarylcubane **3d**

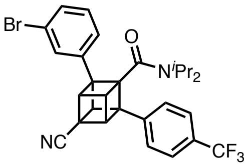

3d was prepared according to General Procedure 3 with **2b'** (60.1 mg, 0.15 mmol) and 1-bromo-3-iodobenzene (152 μL, 1.2 mmol) as a white solid. Purification by silica gel chromatography (EtOAc/$CHCl_3$ = 1/10) and then PTLC (EtOAc/Toluene = 1/8). **3d** (20.7 mg, 0.037 mmol, 24.8%) was obtained by recrystallization from n-hexane/THF. ^{1}H NMR (600 MHz, CD_2Cl_2) δ 7.68 (d, J = 8.2 Hz, 2H), 7.42–7.45 (m, 1H), 7.36–7.40 (m, 3H), 7.30 (t, J = 7.9 Hz, 1H), 7.19 (d, J = 7.6 Hz, 1H), 4.97 (t, J = 2.6 Hz, 1H), 4.45–4.52 (m, 3H), 2.97 (sep, J = 6.5 Hz, 1H), 2.72 (sep, J = 6.5 Hz, 1H), 1.15–1.20 (m, 6H), 0.21 (d, J = 6.5 Hz, 3H), 0.11 (d, J = 6.5 Hz, 3H) ppm. ^{13}C NMR (151 MHz, $CDCl_3$) δ 165.3, 141.5, 139.7, 131.1, 130.8, 130.2 (q, J = 33.3 Hz), 128.8, 126.2, 126.1 (d, J = 2.9 Hz), 124.3, 124.0 (q, J = 271.8 Hz), 123.5, 117.9, 67.9, 59.2, 59.1, 53.3, 53.2, 50.4, 49.6, 46.2, 41.8, 34.0, 20.5, 20.3, 20.3 ppm. HR-MS (ESI-MS, positive) : m/z = 555.1255 calcd for $C_{29}H_{27}N_2OBrF_3$: 555.1253 $[M + H]^+$.

Diarylcubane **3e**

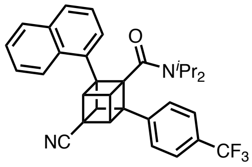

3e was prepared according to General Procedure 3 with **2b'** (80.1 mg, 0.20 mmol) and 1-iodonaphthalene (234 μL, 1.6 mmol) as a white solid (the reaction time for cross coupling : 24 h). Purification by silica gel chromatography (EtOAc/$CHCl_3$ = 1/10) and then GPC. **3e** (28.0 mg, 0.053 mmol, 27%) was obtained by recrystallization from n-hexane/THF in two crops. ^{1}H NMR (600 MHz, $CDCl_3$) δ 7.87 (d, J = 7.6 Hz, 1H), 7.79 (d, J = 8.2 Hz, 1H), 7.66 (d, J = 8.2 Hz, 2H), 7.48–7.57 (m, 4H), 7.41 (d, J = 8.2 Hz, 2H), 7.34 (d, J = 6.9 Hz, 1H), 5.36 (s, 1H), 4.95 (t, J = 5.5 Hz, 1H), 4.52–4.55 (m, 1H), 4.38–4.41 (m, 1H), 2.60–2.71 (m, 2H), 1.11 (d, J = 6.9 Hz, 3H), 0.74 (d, J = 6.9 Hz, 3H), −0.27 (d, J = 6.2 Hz, 3H), −0.59 (d, J = 6.9 Hz, 3H) ppm. ^{13}C NMR (151 MHz, $CDCl_3$) δ 166.1, 141.5, 134.1, 133.8, 131.1, 130.2 (q, J = 33.3 Hz),

129.1, 129.0, 127.0, 126.6, 126.3, 126.0 (d, J = 2.9 Hz), 125.3, 124.6, 124.1 (q, J = 273.2 Hz), 124.0, 118.3, 69.1, 61.8, 58.5, 53.2, 53.0, 49.7, 48.9, 45.8, 41.0, 34.3, 20.3, 20.1, 19.4 ppm. HR-MS (ESI-MS, positive) : m/z = 527.2302 calcd for $C_{33}H_{30}N_2OF_3$: 527.2305 $[M + H]^+$.

<u>Diarylcubane **3f**</u>

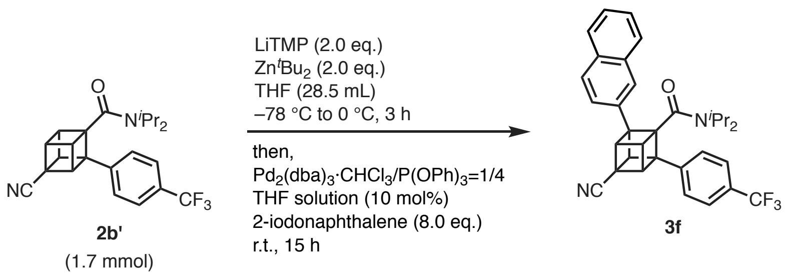

A 200-mL Schlenk tube equipped with J. Young® O-ring tap containing a magnetic stirring bar was charged with **2b'** (680.8 mg, 1.7 mmol) and THF (8.5 mL) under N_2 atmosphere. To the mixture, TMPZn^{t}Bu$_2$Li solution (0.17 M in THF, 20 mL, 3.4 mmol, 2.0 equiv) was added at −78 ºC and the reaction mixture was stirred at 0 ºC for 3 h. $Pd_2(dba)_3$•$CHCl_3$/$P(OPh)_3$ solution ($Pd_2(dba)_3$•$CHCl_3$:0.050 M in THF) was prepared from $Pd_2(dba)_3$•$CHCl_3$ (207.2 mg, 0.20 mmol), triphenyl phosphite (420 μL, 1.6 mmol) and THF (4.0 mL). To the reaction mixture, $Pd_2(dba)_3$•$CHCl_3$/$P(OPh)_3$ solution ($Pd_2(dba)_3$•$CHCl_3$:0.050 M in THF, 3.4 mL, 0.17 mmol, 10 mol%) and 2-iodonaphthalene (3.4 M in THF, 4.0 mL, 13.6 mmol, 8.0 equiv) were added and the solution was stirred for 15 h at room temperature. Then the reaction mixture was quenched with 0.5 N aq. HCl. Then resulting mixture was extracted with EtOAc three times. The organic extracts was washed with H_2O and brine, dried over Na_2SO_4, filtered and concentrated. The residue was then purified by silica gel chromatography (eluent EtOAc/*n*-hexane 0:100 to 25:75 with slow gradient). Crystallization from *n*-hexane/EtOAc afforded 473 mg of white crystals. The residue from the mother liquor was flash chromatographed (eluent EtOAc/*n*-hexane 0:100 to 40:60 with slow gradient) and concentrated. The residue was then filtered on a pad of activated carbon using *n*-hexane/EtOAc 1:1 as eluent. The filtrate was concentrated in vacuo and recrystallized from *n*-hexane/^{i}PrOH to give another 98 mg, total 571 mg (64 %). ^{1}H NMR (600 MHz, $CDCl_3$) δ 7.87 (d, J = 8.2 Hz, 1H), 7.81–7.86 (m, 2H), 7.67 (d, J = 8.2 Hz, 2H), 7.64 (s, 1H), 7.48–7.54 (m, 2H), 7.40 (d, J = 8.2 Hz, 2H), 7.32 (dd, J = 8.6, 1.7 Hz, 1H), 5.24 (t, J = 2.6 Hz, 1H), 4.68 (t, J = 5.5 Hz, 1H), 4.42–4.46 (m, 1H), 4.38–4.41 (m, 1H), 2.88 (sep, J = 6.5 Hz, 1H), 2.72 (sep, J = 6.5 Hz, 1H), 1.18–1.23c (m, 6H), −0.16 (d, J = 6.5 Hz, 3H), −0.22 (d, J = 6.5 Hz, 3H) ppm. ^{13}C NMR (151 MHz, $CDCl_3$) δ 165.8, 141.8, 134.8, 133.4, 132.8, 130.1 (q, J = 33.3 Hz), 129.3, 128.0, 127.9, 127.0, 126.6, 126.3, 126.0 (d, J = 2.9 Hz), 124.3, 124.1 (q, J = 271.8 Hz), 123.5, 118.2, 68.0, 60.2, 59.2,

53.5, 53.2, 50.2, 49.5, 46.0, 41.7, 33.9, 20.5, 20.3, 20.3, 20.2 ppm. HR-MS (ESI-MS, positive) : *m/z* = 527.2304 calcd for $C_{33}H_{30}N_2OF_3$: 527.2305 $[M + H]^+$.

Diarylcubane **3x**

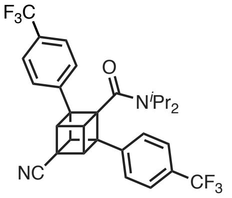

3x was prepared according to General Procedure 3 with **2b'** (200.2 mg, 0.50 mmol), THF (2.5 mL) and 4-iodobenzotrifluoride (580 μL, 4.0 mmol) as a pale yellow solid (the reaction time for cross coupling : 15 h). Purification by silica gel chromatography (eluent $CHCl_3$/EtOAc 10:0 to 10:1 with slow gradient). **3x** (156.1 mg, 0.29 mmol, 57%) was obtained by recrystallization from *n*-hexane/THF. ^{1}H NMR (600 MHz, $CDCl_3$) δ 7.67 (d, J = 8.2 Hz, 4H), 7.35 (d, J = 8.2 Hz, 4H), 5.08–5.10 (m, 1H), 4.63 (t, J = 5.5 Hz, 1H), 4.41–4.43 (m, 2H), 2.96 (sep, J = 6.9 Hz, 1H), 2.66 (sep, J = 6.2 Hz, 1H), 1.21 (d, J = 6.2 Hz, 6H), 0.02 (d, J = 6.9 Hz, 6H) ppm. ^{13}C NMR (151 MHz, $CDCl_3$) δ 165.3, 141.3, 130.2 (q, J = 33.3 Hz), 126.2, 126.1 (d, J = 2.9 Hz), 124.0 (q, J = 271.8 Hz), 117.8, 67.9, 59.2, 53.0, 50.9, 49.3, 46.2, 42.2, 34.1, 20.4, 20.3 ppm. HR-MS (ESI-MS, positive) : *m/z* = 567.1844 calcd for $C_{30}H_{26}N_2NaOF_6$: 567.1842 $[M + Na]^+$.

Synthesis of triarylcubane **4a** (General Procedure 4)

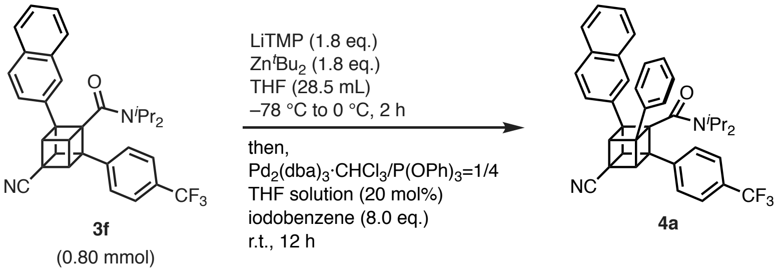

A 100-mL Schlenk tube equipped with J. Young® O-ring tap containing a magnetic stirring bar was charged with **3f** (421.3 mg, 0.80 mmol) and THF (4.0 mL) under N_2 atmosphere. To the mixture, TMPZn^{t}Bu$_2$Li solution (0.17 M in THF, 8.5 mL, 1.44 mmol, 1.8 equiv) was added at −78 ºC and the reaction mixture was stirred at 0 ºC for 2 h. $Pd_2(dba)_3$•$CHCl_3$/ $P(OPh)_3$ solution ($Pd_2(dba)_3$•$CHCl_3$:0.050 M in THF) was prepared from $Pd_2(dba)_3$•$CHCl_3$ (207.2 mg, 0.20 mmol), triphenyl phosphite (420 μL, 1.6 mmol) and THF (4.0 mL). To the reaction mixture, $Pd_2(dba)_3$•$CHCl_3$/$P(OPh)_3$ solution ($Pd_2(dba)_3$•$CHCl_3$:0.050 M in THF, 3.2 mL, 0.16 mmol, 20 mol%) and iodobenzene (715 μL, 6.4 mmol, 8.0 equiv) were added and the solution was stirred for 12 h at room temperature. Then the reaction mixture was

quenched with aq. NH_4Cl. Then resulting mixture was extracted with $CHCl_3$ three times. The organic extracts were dried over Na_2SO_4, and concentrated. The residue was then purified by silica gel chromatography ($CHCl_3$ only) and then PTLC (CH_2Cl_2 only). **4a** (136.4 mg, 0.23 mmol, 28%) was obtained as a white solid by recrystallization from CS_2. ^{1}H NMR (600 MHz, $CDCl_3$) δ 7.92 (d, J = 8.2 Hz, 1H), 7.86 (dd, J = 12.0, 7.9 Hz, 2H), 7.73 (d, J = 7.9 Hz, 2H), 7.70 (s, 1H), 7.42–7.57 (m, 7H), 7.31–7.39 (m, 3H), 5.07 (t, J = 2.6 Hz, 1H), 4.69 (td, J = 3.0, 2.3 Hz, 2H), 2.79 (sep, J = 6.5 Hz, 1H), 2.71 (sep, J = 6.2 Hz, 1H), 1.05–1.11 (m, 6H), −0.28 (d, J = 6.5 Hz, 3H), −0.36 (d, J = 6.2 Hz, 3H) ppm. ^{13}C NMR (151 MHz, $CDCl_3$) δ 164.9, 142.4, 137.9, 135.2, 133.5, 132.8, 129.5 (q, J = 31.8 Hz), 129.3, 128.1, 128.0, 128.0, 126.9, 126.8, 126.5, 125.8, 125.8 (d, J = 2.9 Hz), 124.5, 124.3 (q, J = 271.8 Hz), 123.8, 118.1, 72.4, 57.4, 57.4, 56.0, 56.0, 55.9, 52.7, 49.0, 45.8, 30.9, 20.5, 20.4, 20.11, 20.08 ppm. HR-MS (ESI-MS, positive) : m/z = 625.2447 calcd for $C_{39}H_{33}N_2NaOF_3$: 625.2437 $[M + Na]^+$.

Triarylcubane **4b**

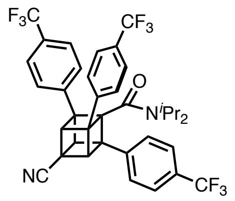

4b was prepared according to General Procedure 4 with **3x** (136.1 mg, 0.25 mmol) and 4-iodobenzotrifluoride (290 μL, 2.0 mmol) as a white solid (TMPZn^{t}Bu$_2$Li : 2.0 equiv). Purification by silica gel chromatography (eluent $CHCl_3$/ n-hexane 1:1 to 1:0 with slow gradient) and then PTLC (EtOAc/CS_2 = 1/10). **4b** (71.6 mg, 0.10 mmol, 42%) was obtained by recrystallization from CS_2. ^{1}H NMR (600 MHz, $CDCl_3$) δ 7.73 (d, J = 7.9 Hz, 6H), 7.44 (d, J = 7.9 Hz, 6H), 4.80 (s, 3H), 2.85 (sep, J = 6.9 Hz, 1H), 2.58 (sep, J = 6.5 Hz, 1H), 1.08 (d, J = 6.9 Hz, 6H), −0.21 (d, J = 6.5 Hz, 6H) ppm. ^{13}C NMR (151 MHz, $CDCl_3$) δ 164.1, 141.4, 130.3 (q, J = 33.3 Hz), 126.4, 126.2 (d, J = 4.3 Hz), 124.0 (q, J = 272.0 Hz), 117.3, 72.2, 56.7, 54.8, 48.9, 46.1, 31.2, 20.3, 20.1 ppm. HR-MS (ESI-MS, positive) : m/z = 689.2210 calcd for $C_{37}H_{30}N_2OF_9$: 689.2009 $[M + H]^+$.

Triarylcubane **4c**

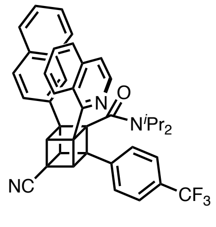

4c was prepared according to General Procedure 4 with **3f** (105.3 mg, 0.20 mmol), THF (2.4 mL) and 1-iodoisoquinoline (408.1 mg, 1.6 mmol) as a white solid (TMPZn^{t}Bu$_2$Li : 2.0 equiv,

the reaction time for orthometalation : 2.5 h). Purification by silica gel chromatography ($EtOAc/CHCl_3$ = 1/10) and then PTLC (EtOAc/toluene = 1/12). **4c** (6.3 mg, 9.6 µmol, 5%) was obtained by recrystallization from *n*-hexane/MeTHF. ^{1}H NMR (600 MHz, $CDCl_3$) δ 8.58 (d, *J* = 5.5 Hz, 1H), 7.96–8.01 (m, 2H), 7.86–7.93 (m, 4H), 7.79 (d, *J* = 7.9 Hz, 2H), 7.67–7.72 (m, 3H), 7.62 (d, *J* = 5.5 Hz, 1H), 7.60 (dd, *J* = 8.4, 1.5 Hz, 1H), 7.50–7.57 (m, 3H), 5.62 (t, *J* = 2.4 Hz, 1H), 5.21 (t, *J* = 2.6 Hz, 1H), 4.53 (t, *J* = 2.7 Hz, 1H), 2.83 (sep, *J* = 6.5 Hz, 1H), 2.58 (sep, *J* = 6.9 Hz, 1H), 0.87 (d, *J* = 6.9 Hz, 3H), 0.70 (d, *J* = 6.5 Hz, 3H), −0.48 – −0.54 (m, 6H) ppm. ^{13}C NMR (151 MHz, $CDCl_3$) δ 165.6, 156.5, 142.5, 142.2, 136.4, 135.2, 133.5, 132.9, 130.8, 129.7 (q, *J* = 33.3 Hz), 129.1, 128.1, 128.0, 127.9, 127.8, 127.4, 126.9, 126.6, 126.5, 125.9 (d, *J* = 2.9 Hz), 125.3, 125.1, 124.3 (q, *J* = 271.8 Hz), 124.3, 120.9, 118.0, 73.6, 59.2, 58.3, 58.0, 56.8, 53.6, 51.4, 49.0, 45.7, 32.1, 20.3, 20.2, 19.7, 19.6 ppm. HR-MS (ESI-MS, positive) : *m/z* = 654.2725 calcd for $C_{42}H_{35}N_3OF_3$: 654.2727 $[M + H]^+$.

7. *Transformation of Multiply Arylated Cubanes* [S8], [S9]

6-1. Decarboxylation of triarylcubane **4a**

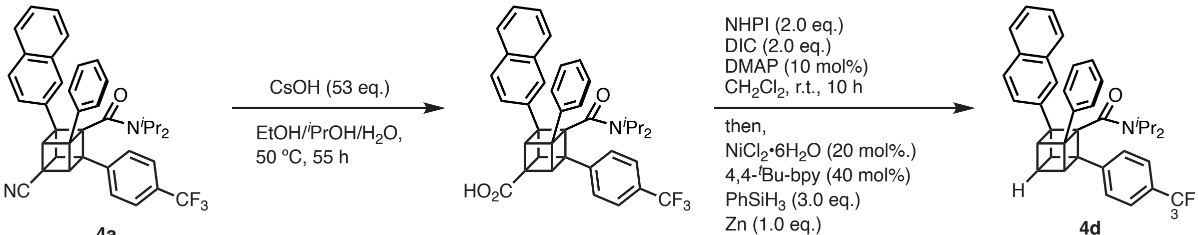

4a (226 µmol) **4d** (45% in 3 steps)

A culture tube was containing a magnetic stirring bar was charged with **4a** (136.4 mg, 0.23 mmol), EtOH (1.1 mL) and ^{i}PrOH (4.2 mL). To the mixture, aq. CsOH (5.0 M, 1.2 mL, 6.0 mmol, 26.5 equiv) was added and the reaction mixture was stirred at 50 ºC. After 19 h stirring, aq. CsOH (5.0 M, 0.60 mL, 3.0 mmol, 13.3 equiv) was added to the mixture and the reaction mixture was stirred at 50 ºC. After 12 h stirring, aq. CsOH (5.0 M, 0.60 mL, 3.0 mmol, 13.3 equiv) and EtOH (0.60 mL) were added to the mixture and the reaction mixture was stirred for 24 h at 50 ºC. Then the reaction mixture was quenched with 2.0 M aq. HCl. Then resulting mixture was extracted with $CHCl_3$ three times. The organic extracts were dried over Na_2SO_4, and concentrated. The residue was used as triarylcubane carboxylic acid without further purification.

A 50 mL round-bottom flask containing a magnetic stirring bar was charged with triarylcubane carboxylic acid (101.3 mg, 0.163 mmol), *N*-hydroxyphthalimide (53.2 mg, 0.326 mmol, 2.0 equiv), DMAP (2.0 mg, 16 µmol, 10 mol%) and suspended in 4.0 mL dry DCM under N_2 atmosphere. After addition of *N*,*N*'-diisopropylcarbodiimide (50 µL, 0.326

mmol, 2.0 equiv), the reaction mixture was stirred for 10 h in the dark at room temperature. The reaction mixture was filtered through celite and the solvent removed in vacuo. The residue was purified by silica gel chromatography (CH_2Cl_2). This was used as redox-active ester cubane without further purification.

A 20-mL Schlenk tube equipped with J. Young® O-ring tap containing a magnetic stirring bar was charged with the redox-active ester cubane (0.134 mmol), Zn metal (8.8 mg, 0.134 mmol, 1.0 equiv) under N_2 atmosphere. THF (0.75 mL) and ^{i}PrOH (75 µL) were added. A solution of $NiCl_2$•$6H_2O$/4,4'-^{t}Bu-bpy (0.10 M in DMF, 0.27 mL, 10 mol% $NiCl_2$•$6H_2O$, 20 mol% 4,4'-^{t}Bu-bpy) and $PhSiH_3$ (50 µL, 0.402 mmol, 3.0 equiv) were added in quick succession. The reaction mixture was stirred for 1 h at 40 ºC. Then, the mixture was quenched with H_2O, sat. aq. NH_4Cl. The mixture was extracted with EtOAc, and the organic extracts were dried over Na_2SO_4. The organic extracts were concentrated on a rotary evaporator. The residue was then purified by silica gel chromatography ($CHCl_3$). **4d** (58.4 mg, 0.10 mmol, 45% in 3 steps) was obtained as a white solid by recrystallization from *n*-hexane/THF.

Triarylcubane **4d**

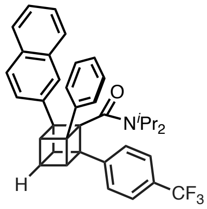

^{1}H NMR (600 MHz, CD_3CN) δ 7.92 (t, *J* = 7.7 Hz, 2H), 7.88 (d, *J* = 7.9 Hz, 1H), 7.82 (s, 1H), 7.73 (d, *J* = 7.9 Hz, 2H), 7.60 (d, *J* = 7.9 Hz, 2H), 7.46–7.54 (m, 3H), 7.38–7.45 (m, 4H), 7.24–7.29 (m, 1H), 4.72–4.75 (m, 1H), 4.44 (dq, *J* = 7.3, 2.2 Hz, 2H), 3.84 (q, *J* = 5.2 Hz, 1H), 2.74–2.83 (m, 2H), 0.95–1.05 (m, 6H), −0.35 (d, *J* = 6.2 Hz, 3H), −0.41 (d, *J* = 6.5 Hz, 3H) ppm. ^{13}C NMR (151 MHz, CD_3CN) δ 167.2, 146.8, 141.6, 139.2, 134.5, 133.2, 129.5, 129.1, 128.6, 128.4 (q, *J* = 31.8 Hz), 128.1, 127.5, 127.3, 127.0, 126.7, 125.9 (d, *J* = 4.3 Hz), 125.8, 125.7 (q, *J* = 272.0 Hz), 125.2, 72.5, 59.0, 58.9, 57.7, 54.0, 53.7, 51.1, 49.1, 45.9, 37.3, 20.6, 20.5, 20.51, 20.49 ppm. HR-MS (ESI-MS, positive) : *m/z* = 578.2665 calcd for $C_{38}H_{35}NOF_3$: 578.2665 $[M + H]^+$.

6-2. Decarboxylative phenylation of diarylcubane **3f**

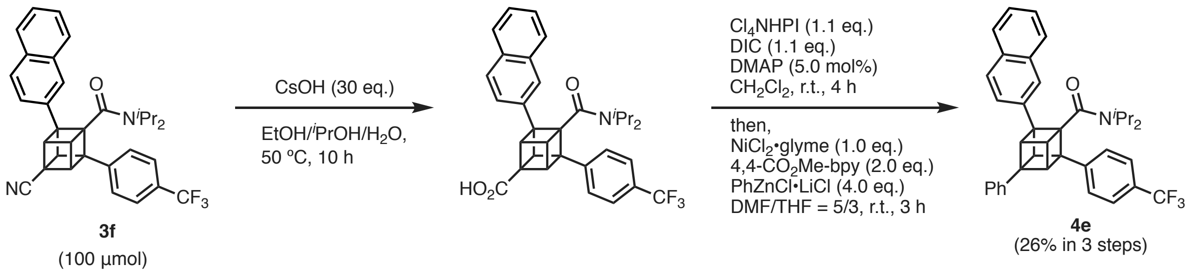

A culture tube was containing a magnetic stirring bar was charged with **3f** (52.7 mg, 0.10 mmol), EtOH (0.60 mL) and ^{i}PrOH (2.1 mL). To the mixture, aq. CsOH (5.0 M, 0.60 mL, 3.0 mmol, 30 equiv) was added and the reaction mixture was stirred for 10 h at 50 ºC. Then the reaction mixture was quenched with 2.0 M aq. HCl. Then resulting mixture was extracted with $CHCl_3$ three times. The organic extracts were dried over Na_2SO_4, and concentrated. The residue was used as diarylcubane carboxylic acid without further purification.

A 50 mL round-bottom flask was containing a magnetic stirring bar was charged with diarylcubane carboxylic acid (40.8 mg, 0.075 mmol), tetrachlorohydroxyisoindoline-1,3-dione (24.7 mg, 0.082 mmol, 1.1 equiv), DMAP (0.47 mg, 3.7 μmol, 5 mol%) and suspended in 2.0 mL dry DCM under N_2 atmosphere. After addition of *N,N*'-diisopropylcarbodiimide (12.8 μL, 0.082 mmol, 1.1 equiv), the reaction mixture was stirred for 4 h in the dark at room temperature. The reaction mixture was filtered through celite and the solvent removed in vacuo. The residue was used as redox-active ester cubane without further purification due to sensibility to moisture and SiO_2.

A 20-mL Schlenk tube equipped with J. Young® O-ring tap containing a magnetic stirring bar was charged with the redox-active ester cubane (0.075 mmol), $NiCl_2$-glyme (16.4 mg, 0.075 mmol, 1.0 equiv) and 4,4'-Dimethoxycarbonyl-2,2'-bipyridine (40.7 mg, 0.15 mmol, 2.0 equiv) under N_2 atmosphere. Finally, the yellow solids were dissolved in DMF (1.0 mL) and stirred under N_2 atmosphere. PhZnCl•LiCl solution (0.50 M in THF) was prepared from LiCl (42.4 mg), PhMgBr solution (3.0 M in Et_2O, 0.35 mL), THF (0.65 mL) and $ZnCl_2$ solution (1.0 M in THF, 1.0 mL). Then the PhZnCl•LiCl solution (600 μL, 0.050 M, 0.30 mmol, 4.0 equiv) was added in one portion and the mixture was stirred for 3 h at room temperature. The reaction mixture was quenched with 2.0 M aq. HCl. Then resulting mixture was extracted with $CHCl_3$ three times. The organic extracts were dried over Na_2SO_4, and concentrated. The residue was then purified by silica gel chromatography ($CHCl_3$). **4e** (14.9 mg, 26 μmol, 26% in 3 steps) was obtained as a white solid by recrystallization from *n*-hexane.

Triarylcubane **4e**

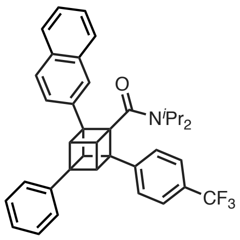

^{1}H NMR (600 MHz, cyclohexane-d_{12}) δ 7.73 (d, J = 8.2 Hz, 1H), 7.69 (dd, J = 12.4, 8.2 Hz, 2H), 7.59 (s, 1H), 7.55 (d, J = 7.6 Hz, 2H), 7.29–7.40 (m, 5H), 7.20 (t, J = 7.9 Hz, 2H), 7.10–7.13 (m, 2H), 7.05–7.09 (m, 1H), 4.84 (s, 1H), 4.53 (t, J = 5.5 Hz, 1H), 4.17–4.20 (m, 1H), 4.10–4.13 (m, 1H), 2.94 (sep, J = 6.2 Hz, 1H), 2.83 (sep, J = 6.8 Hz, 1H), 1.21–1.32 (m, 6H), −0.19–−0.07 (m, 6H) ppm. ^{13}C NMR (151 MHz, cyclohexane-d_{12}) δ 166.6, 145.3, 142.0, 138.2, 134.6, 133.5, 130.2 (q, J = 31.8 Hz), 129.2, 129.0, 128.3, 128.1, 126.8, 126.8, 126.7, 126.1, 126.0 (d, J = 2.9 Hz), 125.5, 124.9 (q, J = 271.8 Hz), 124.8, 124.3, 68.8, 58.4, 57.4, 55.3, 54.7, 54.0, 53.0, 49.2, 46.4, 40.3, 21.0, 20.7, 20.6, 20.5 ppm. HR-MS (ESI-MS, positive) : m/z = 578.2663 calcd for $C_{38}H_{35}NOF_3$: 578.2665 $[M + H]^+$.

6-3. Decarboxylative coupling of triarylcubane **4b**

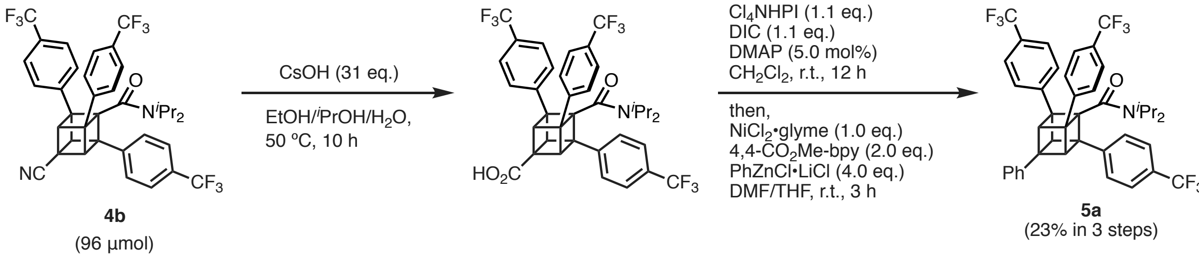

A culture tube was containing a magnetic stirring bar was charged with **4b** (66.3 mg, 0.096 mmol), EtOH (2.1 mL) and ^{i}PrOH (1.0 mL). To the mixture, aq. CsOH (5.0 M, 0.60 mL, 3.0 mmol, 31 equiv) was added and the reaction mixture was stirred for 10 h at 50 ºC. Then the reaction mixture was quenched with 2.0 M aq. HCl. Then resulting mixture was extracted with $CHCl_3$ three times. The organic extracts were dried over Na_2SO_4, and concentrated. The residue was used as triarylcubane carboxylic acid without further purification.

A 50 mL round-bottom flask was containing a magnetic stirring bar was charged with triarylcubane carboxylic acid (61.73 mg, 0.087 mmol), tetrachlorohydroxyisoindoline-1,3-dione (28.9 mg, 0.096 mmol, 1.1 equiv), DMAP (0.53 mg, 4.4 μmol, 5.0 mol%) and suspended in 2.0 mL dry DCM under N_2 atmosphere. After addition of *N*,*N*'-diisopropylcarbodiimide (15 μL, 0.096 mmol, 1.1 equiv), the reaction mixture was stirred for 12 h in the dark at room temperature. The reaction mixture was filtered through celite and the

solvent removed in vacuo. The residue was used as redox-active ester cubane without further purification due to sensibility to moisture and SiO_2.

A 20-mL Schlenk tube equipped with J. Young® O-ring tap containing a magnetic stirring bar was charged with the redox-active ester cubane (0.087 mmol), $NiCl_2$-glyme (19.2 mg, 0.087 mmol, 1.0 equiv) and 4,4'-Dimethoxycarbonyl-2,2'-bipyridine (47.5 mg, 0.174 mmol, 2.0 equiv) under N_2 atmosphere. Finally, the yellow solids were dissolved in DMF (1.0 mL) and stirred under N_2 atmosphere. PhZnCl•LiCl solution (0.50 M in THF) was prepared from LiCl (42.4 mg), PhMgBr solution (3.0 M in Et_2O, 0.35 mL), THF (0.65 mL) and $ZnCl_2$ solution (1.0 M in THF, 1.0 mL). Then the PhZnCl•LiCl solution (700 µL, 0.050 M, 0.35 mmol, 4.0 equiv) was added in one portion and the mixture was stirred for 3 h at room temperature. The reaction mixture was quenched with 2.0 M aq. HCl. Then resulting mixture was extracted with $CHCl_3$ three times. The organic extracts were dried over Na_2SO_4, and concentrated. The residue was then purified by silica gel chromatography (CH_2Cl_2) and PTLC (CH_2Cl_2). **5x** (16.6 mg, 22 µmol, 23% in 3 steps) was obtained as a white solid by recrystallization from CS_2.

Tetraarylcubane **5a**

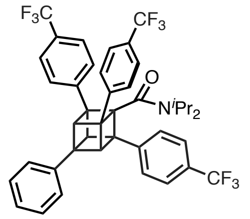

^{1}H NMR (600 MHz, $CDCl_3$) δ 7.69 (d, J = 7.9 Hz, 6H), 7.49 (d, J = 7.9 Hz, 6H), 7.34 (t, J = 7.7 Hz, 2H), 7.23 (t, J = 7.4 Hz, 1H), 7.15 (dd, J = 8.4, 1.2 Hz, 2H), 4.59 (s, 3H), 2.89 (sep, J = 6.5 Hz, 1H), 2.76 (sep, J = 6.5 Hz, 1H), 1.14 (d, J = 6.5 Hz, 6H), −0.18 (d, J = 6.5 Hz, 6H) ppm. ^{13}C NMR (151 MHz, $CDCl_3$) δ 165.7, 143.8, 140.0, 129.4 (q, J = 31.8 Hz), 128.9, 127.1, 126.5, 125.9 (d, J = 4.3 Hz), 125.2, 124.3 (q, J = 271.8 Hz), 72.3, 56.3, 54.5, 50.0, 48.6, 45.9, 20.5, 20.3 ppm. HR-MS (ESI-MS, positive) : m/z = 762.2391 calcd for $C_{42}H_{34}NNaOF_9$: 762.2389 $[M + Na]^+$.

7. *X-ray Crystallographic Analysis*

Details of the crystal data and the intensity data collection parameters for **2a**, **2e**, **2f**, **2k**, **2p**, **3a**, **4b** and **5a** are listed in Table S2–4 and Figure S1–8. In each case, a suitable crystal was mounted with mineral oil on a glass fiber and transferred to the goniometer of a Rigaku PILATUS diffractometer. Graphite-monochromated Mo Kα radiation (λ = 0.71073 Å) was used. Cell parameters were determined and refined, and raw frame data were integrated using CrysAlis^{Pro} (Agilent Technologies, 2010). The structures were solved by direct methods with SIR-97,[S10] SHELXS,[S11] or SHELXT[S12] and refined by full-matrix least-squares techniques against F^2 (SHELXL-2016/6 or SHELXL-2018/3)[S11] with Yadokari-XG program[S13] or Olex2[S14] software package. The intensities were corrected for Lorentz and polarization effects. The non-hydrogen atoms were refined anisotropically. Hydrogen atoms were placed using AFIX instructions.

Table S2. Crystallographic data and refinement details for **2a**, **2e**, **2f.**

	**2a**	**2e**	**2f**
CCDC Number	1993519	1993515	1993518
formula	$C_{29}H_{37}N_3O_2$	$C_{68}H_{84}N_4O_4$	$C_{31}H_{42}N_2O_4$
fw	459.61	1021.39	506.66
T (K)	123(2)	123(2)	123(2)
λ (Å)	0.71073	0.71073	0.71073
cryst syst	Orthorhombic	Triclinic	Monoclinic
space group	*Pbca*	*P*-1	$P2_1/n$
a (Å)	12.4001(8)	13.5526(5)	12.9747(2)
b (Å)	25.3104(17)	14.1690(5)	12.3749(2)
c (Å)	16.4161(9)	17.1029(5)	17.7595(3)
α (deg)	90	103.450(3)	90
β (deg)	90	107.340(3)	101.9476(17)
γ (deg)	90	103.234(3)	90
V (Å^3)	5152.2(6)	2880.96(18)	2789.71(8)
Z	8	2	4
D_{calc} (g/cm^3)	1.185	1.177	1.206
μ (mm^{-1})	0.075	0.072	0.079
F(000)	1984	1104	1096
cryst size (mm)	0.20 × 0.05 × 0.03	0.15 × 0.05 × 0.02	0.20 × 0.20 × 0.10
θ range (deg)	2.481–24.998	2.347–24.998	2.344–24.996
reflns collected	35337	31848	29403
indep reflns/R_{int}	4538/0.0930	10091/0.0742	4911/0.0214
params	315	701	343
GOF on F^2	1.017	1.061	1.035
R_1, wR_2 [I>2σ(I)]	0.0484, 0.0932	0.0768, 0.1775	0.0343, 0.0866
R_1, wR_2 (all data)	0.0940, 0.1094	0.1427, 0.2124	0.0374, 0.0887

Table S3. Crystallographic data and refinement details for **2k**, **2p**, **3a.**

	**2k**	**2p**	**3a**
CCDC Number	1993517	1993513	1993512
formula	$C_{32}H_{40}N_2O_2$	$C_{31}H_{39}N_3O_2$	$C_{30}H_{26}F_3N_3O$
fw	484.66	485.65	501.54
T (K)	123(2)	123(2)	123(2)
λ (Å)	0.71073	0.71073	0.71073
cryst syst	Monoclinic	Triclinic	Monoclinic
space group	$P2_1/n$	P-1	$P2_1/n$
a (Å)	10.4974(4)	8.9740(2)	14.0809(7)
b (Å)	24.3000(6)	11.7370(3)	10.9917(4)
c (Å)	11.3737(4)	14.0121(3)	17.1659(8)
α (deg)	90	113.955(2)	90
β (deg)	109.196(4)	97.7538(17)	108.846(5)
γ (deg)	90	91.349(2)	90
V (Å^3)	2739.97(17)	1331.40(6)	2514.4(2)
Z	4	2	4
D_{calc} (g/cm^3)	1.175	1.211	1.325
μ (mm^{-1})	0.073	0.076	0.097
F(000)	1048	524	1048
cryst size (mm)	0.10 × 0.05 × 0.01	0.25 × 0.20 × 0.10	0.10 × 0.10 × 0.10
θ range (deg)	2.441–24.998	2.299–24.999	1.634–24.999
reflns collected	29817	14591	13618
indep reflns/R_{int}	4822/0.0292	4665/0.0192	4418/0.0510
params	333	333	375
GOF on F^2	1.026	1.028	1.106
R_1, wR_2 [I>2σ(I)]	0.0347, 0.0817	0.0365, 0.0927	0.0615, 0.1401
R_1, wR_2 (all data)	0.0432, 0.0866	0.0402, 0.0949	0.0774, 0.1466

Table S4. Crystallographic data and refinement details for **4b**, **5a.**

	**4b·C_6H_6**	**5a**
CCDC Number	1993516	1993514
formula	$C_{43}H_{35}F_9N_2O$	$C_{42}H_{34}$ F_9NO
fw	776.73	739.70
T (K)	123(2)	123(2)
λ (Å)	0.71073	0.71073
cryst syst	Triclinic	Orthorhombic
space group	*P*-1	*Pccn*
a (Å)	9.7571(1)	37.632(1)
b (Å)	10.1037(1)	14.8891(4)
c (Å)	19.2300(3)	13.0219(4)
α (deg)	86.600(1)	90
β (deg)	88.612(1)	90
γ (deg)	81.412(1)	90
V (Å^3)	1870.97(4)	7296.3(4)
Z	2	8
D_{calc} (g/cm^3)	1.361	1.347
μ (mm^{-1})	0.112	0.112
F(000)	792	3056
cryst size (mm)	0.10 × 0.10 × 0.10	0.15 × 0.10 × 0.05
θ range (deg)	2.348–29.766	2.123–27.888
reflns collected	37233	27734
indep reflns/R_{int}	9620/0.0286	7329/0.0503
params	583	510
GOF on F^2	1.059	1.071
R_1, wR_2 [I>2σ(I)]	0.0465, 0.1209	0.0697, 0.1559
R_1, wR_2 (all data)	0.0610, 0.1286	0.1062, 0.1699

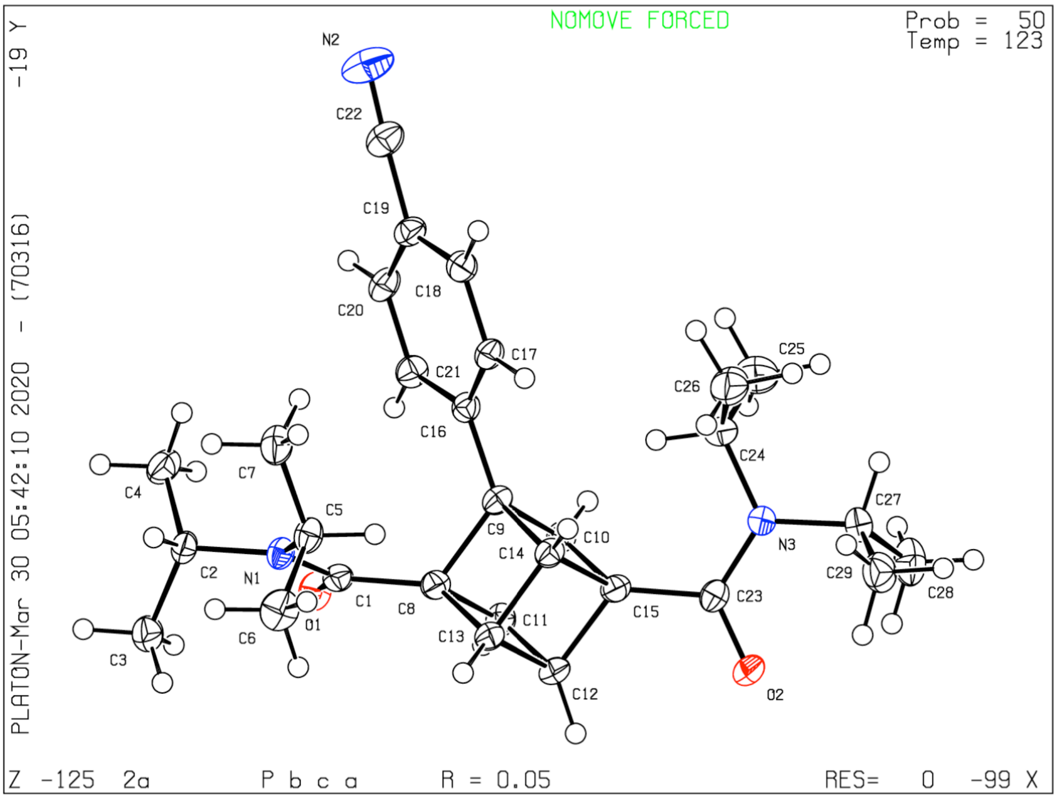

Figure S1. X-ray single crystal structure of **2a**.

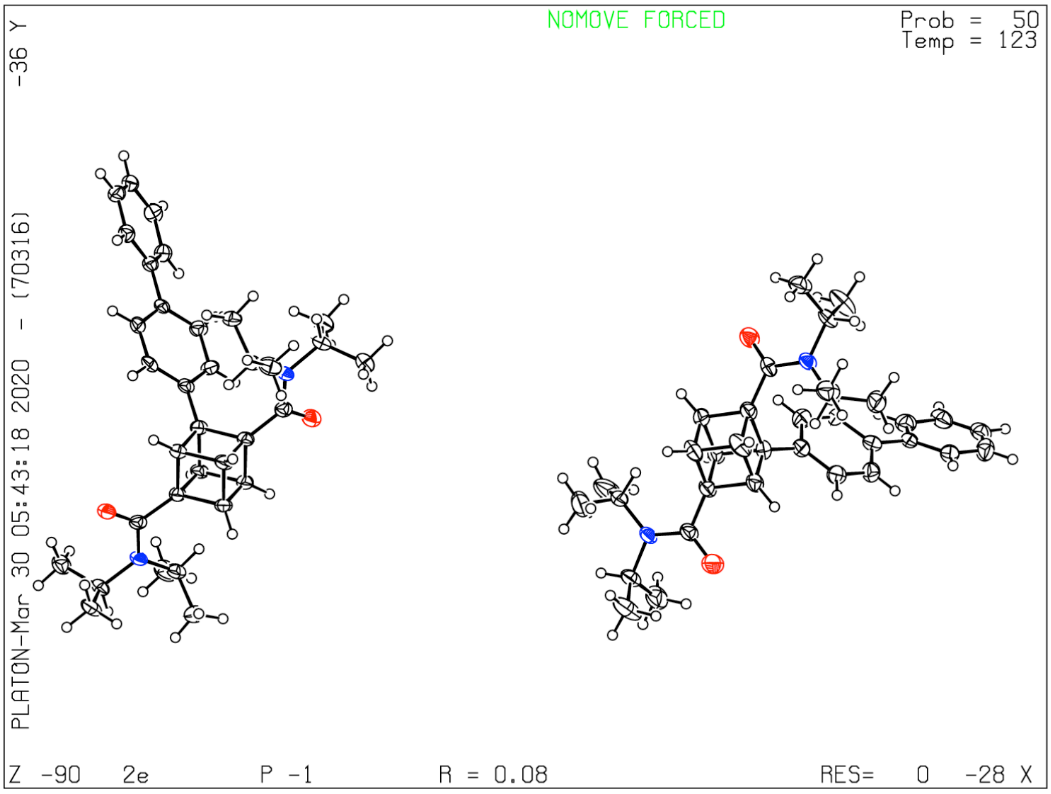

Figure S2. X-ray single crystal structure of **2e**.

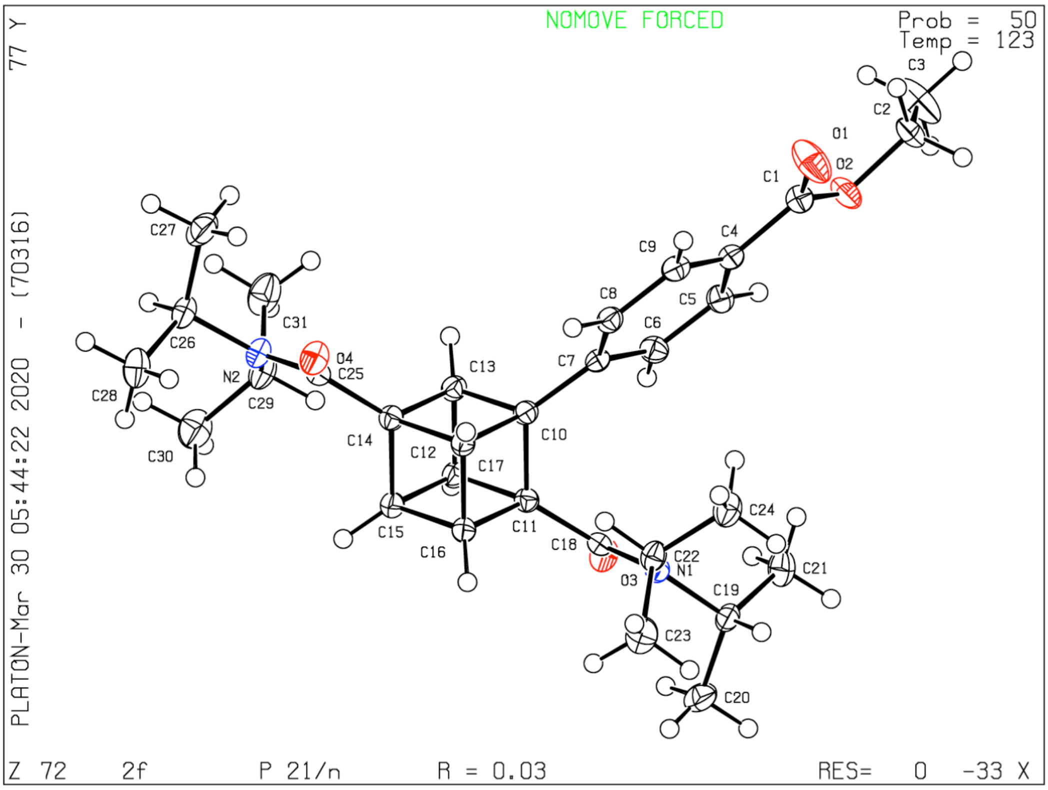

Figure S3. X-ray single crystal structure of **2f**.

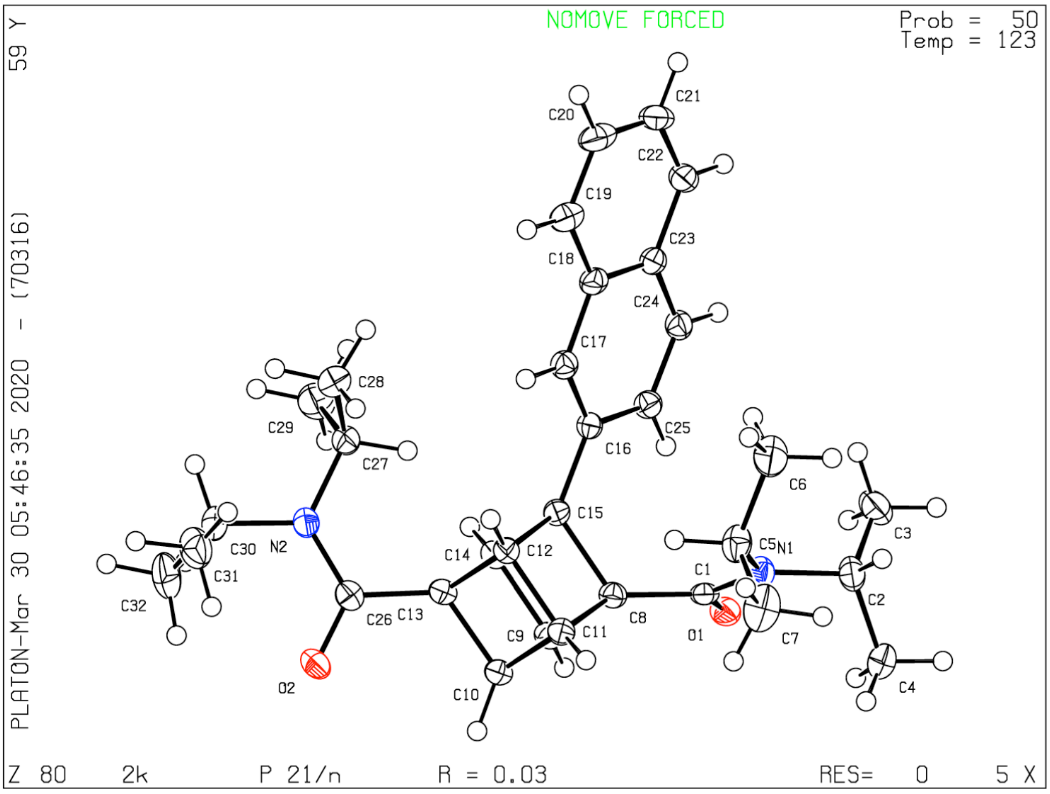

Figure S4. X-ray single crystal structure of **2k**.

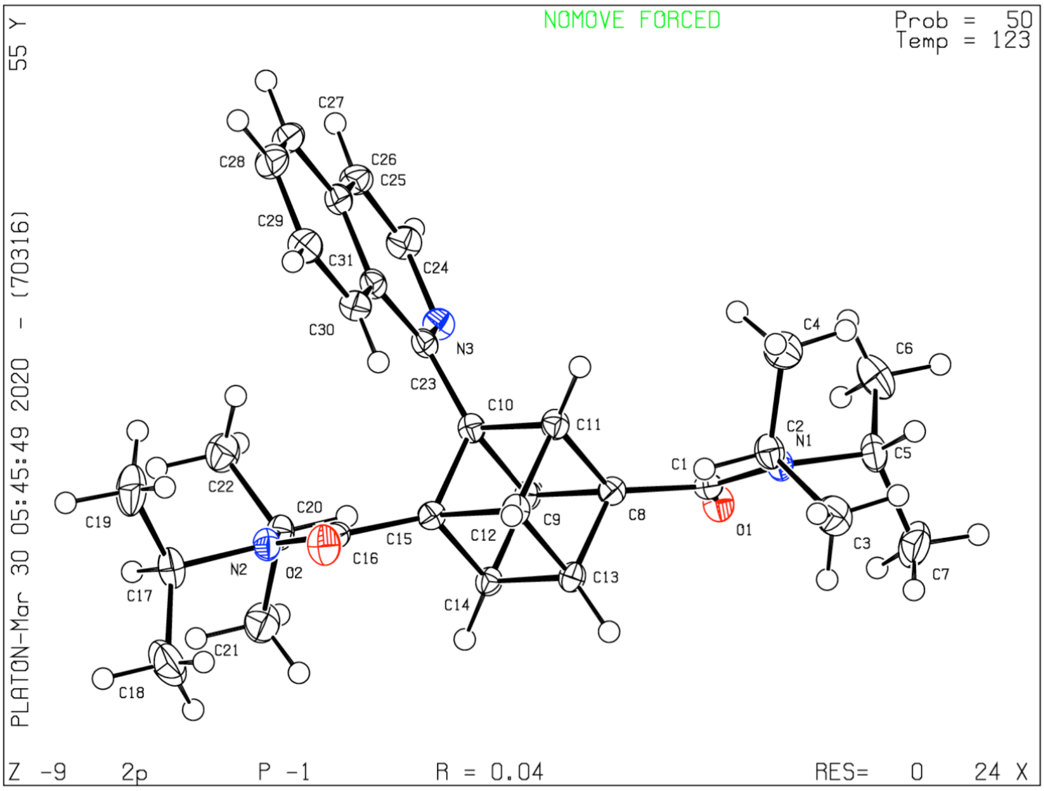

Figure S5. X-ray single crystal structure of **2p**.

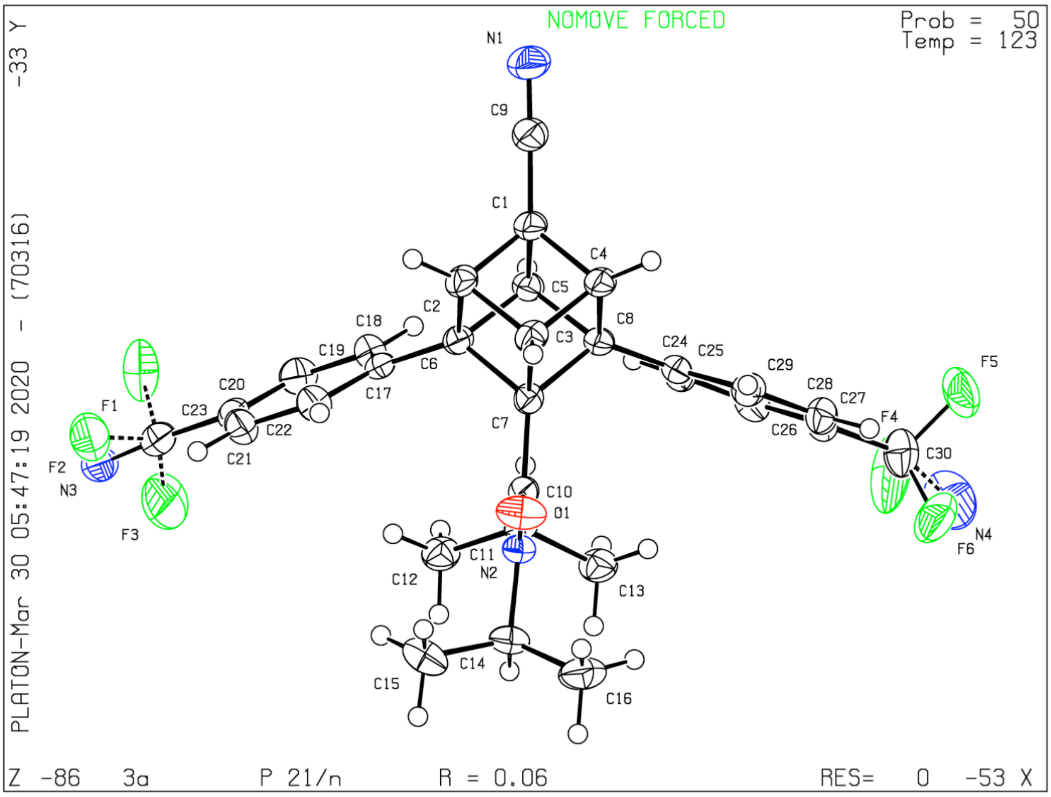

Figure S6. X-ray single crystal structure of **3a**.

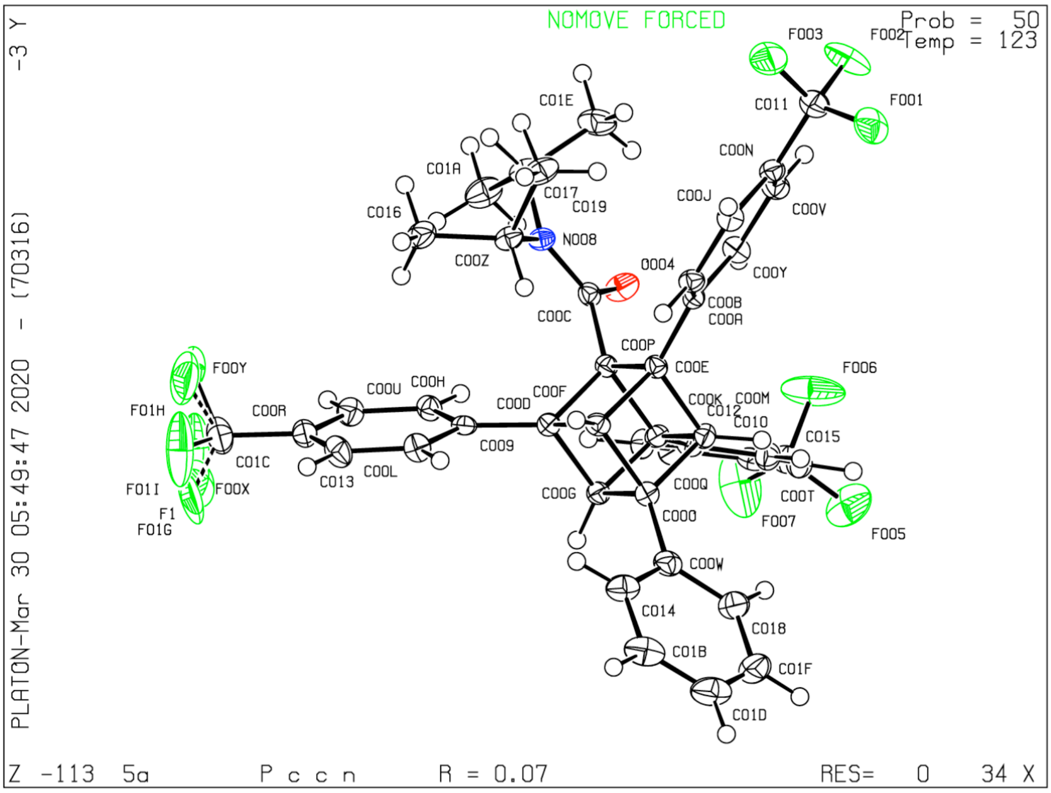

Figure S7. X-ray single crystal structure of **4b**.

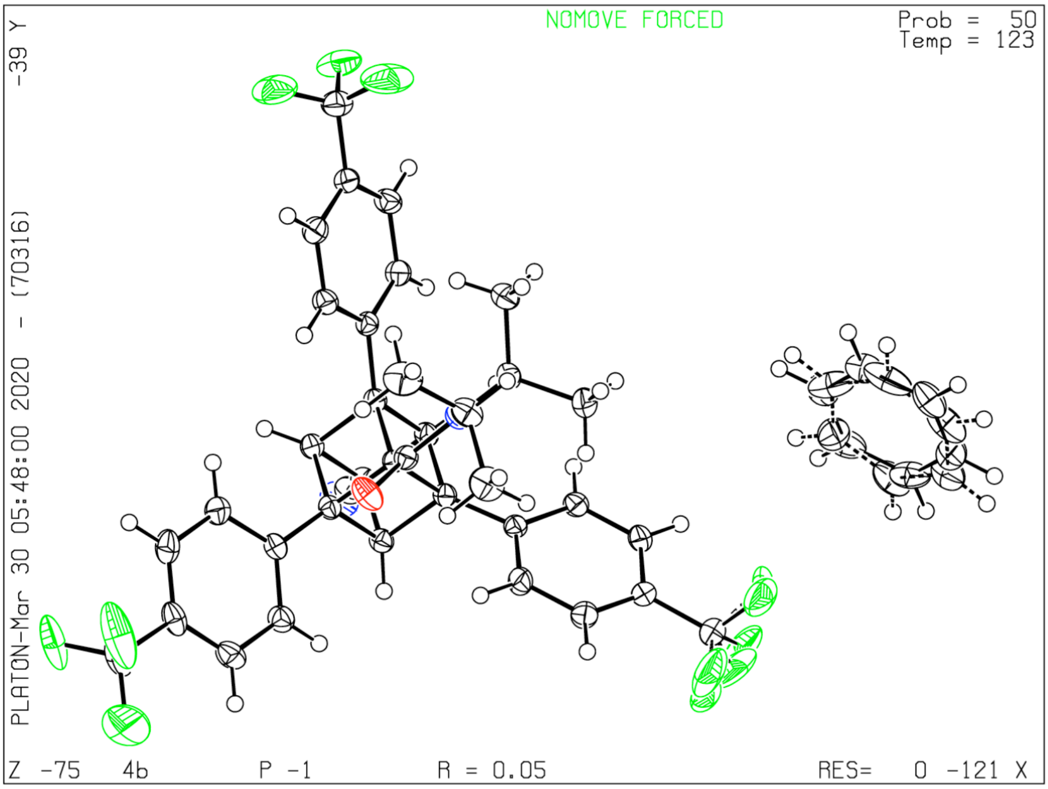

Figure S8. X-ray single crystal structure of **5a**.

8. *HPLC Chart*

HPLC analysis was conducted on a Shimadzu Prominence 2000 instrument equipped with equipped with a CHIRALPAK® ID-3 column (eluent: n-hexane/^{i}PrOH = 90/10, 1.0 mL·s^{-1}, 35 °C, Detector: PDA Ch1 254 nm 4 nm).

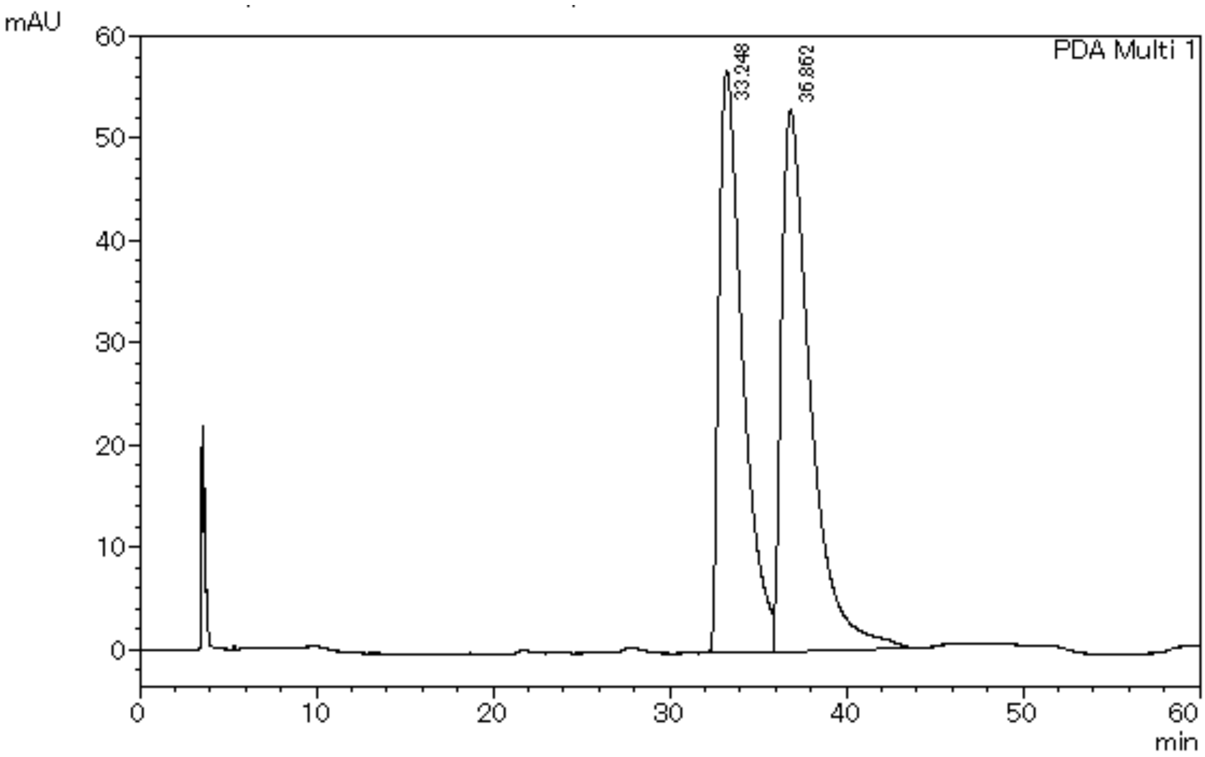

Figure S9. Chromatogram of **3a**.

Table S5. Peak table of **3a**.

Peak #	Retention Time [min]	Area [mAU*s]	Height [mAU]	Area %
1	33.248	5342.042	56.959	46.994
2	36.862	6025.430	52.900	53.006

HPLC analysis was conducted on a Shimadzu Prominence 2000 instrument equipped with equipped with a CHIRALPAK® ID-3 column (eluent: n-hexane/^{i}PrOH = 95/5.0, 1.0 mL·s^{-1}, 35 °C, Detector: PDA Ch1 254 nm 4 nm).

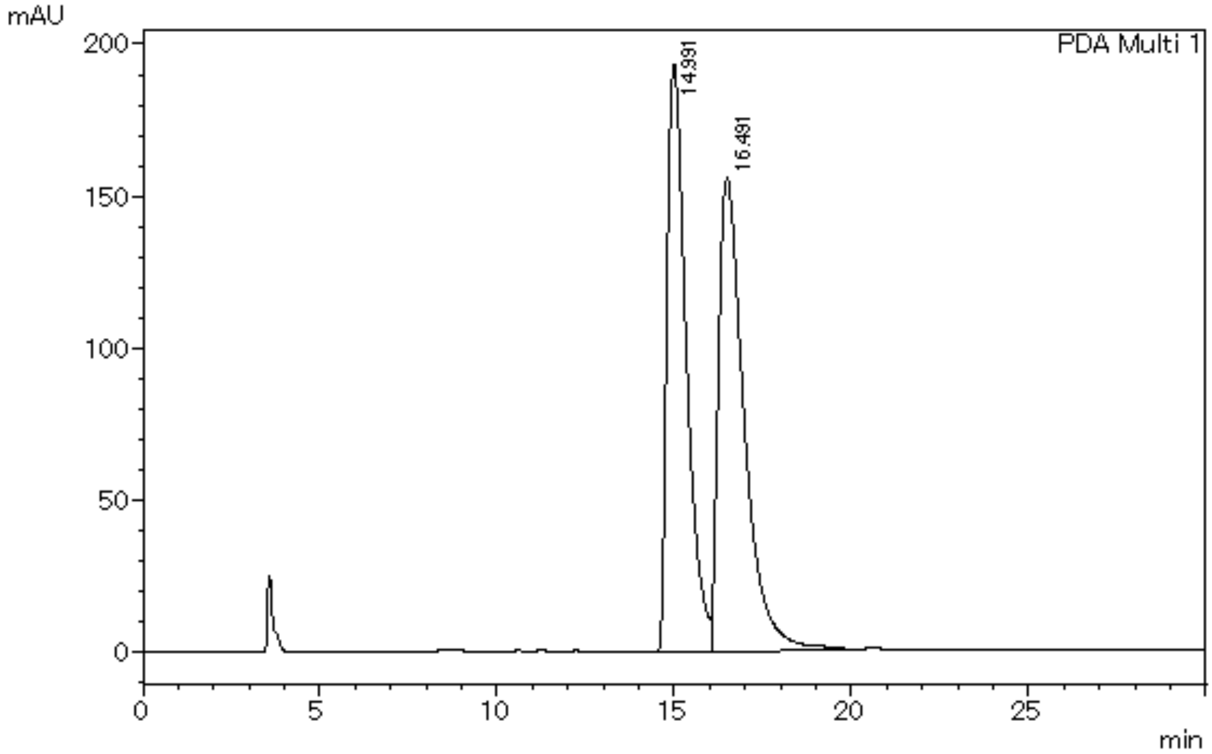

Figure S10. Chromaogram of **3f**.

Table S6. Peak table of **3f**.

Peak #	Retention Time [min]	Area [mAU*s]	Height [mAU]	Area %
1	14.991	7365.028	192.240	48.416
2	16.491	7847.087	154.854	51.584

9. *Reference*

[1] T. Ukai, H. Kawazura, Y. Ishii, J. J. Bonnet, J. A. Ibers, *J. Organometallic Chem.* **1974**, *65*, 253.

[2] Y. Sawamura, H. Nakatsuji, A Sakakura, K. Ishihara, *Chem. Sci.* **2013**, *4*, 4181.

[3] G. Özturk, M. Förstel, Y. Ergun, S. Alp, W. Rettig, *J. Fluoresc.* **2008**, *18*, 1007.

[4] C. Wallinder, M. Botros, U. Rosenström, M.-O. Guimond, H. Beaudry, F. Nyberg, N. Gallo-Payet, A. Hallberg, M. Alterman, *Bioorg. Med. Chem.* **2008**, *16*, 6841.

[5] M. Jean, J. Renault, P. Uriac, M. Capet, P. van de Weghe, *Org. Lett.* **2007**, *9*, 3623.

[6] P. E. Eaton, Y. Xiong, J. P. Zhou, *J. Org. Chem.* **1992**, *57*, 4277.

[7] Y. Kondo, M. Shilai, M. Uchiyama, T. Sakamoto, *J. Am. Chem. Soc.* **1999**, *121*, 3539.

[8] S. S. R. Bernhard, G. M. Locke, S. Plunkett, A. Meindl, K. J. Flanagan, M. O. Senge, *Chem. -Eur. J.* **2018**, *24*, 1026.

[9] T. Qin, L. R. Malins, J. T. Edwards, R. R. Merchant, A. J. E. Novak, J. Z. Zhong, R. B. Mills, M. Yan, C. Yuan, M. D. Eastgate, P. S. Baran, *Angew. Chem., Int. Ed.* **2017**, *56*, 260.

[10] A. Altomare, M. C. Burla, M. Camalli, G. L. Cascarano, C. Giacovazzo, A. Guagliardi, A. G. G. Moliterni, G. Polidori, R. Spagna, SIR97: a New Tool for Crystal Structure Determination and Refinement. *J. Appl. Crystallogr.* **1999**, *32*, 115.

[11] G. M. Sheldrick, *Acta Crystallogr.*, **2015**, *C71*, 3.

[12] G. M. Sheldrick, *Acta Crystallogr.*, **2015**, *A71*, 3.

[13] (a) Yadokari-XG Software for Crystal Structure Analyses. K. Wakita 2001. (b) K. Chizuko, A. Shigehisa, K. Eunsang, Release of Software（Yadokari-XG 2009）for Crystal Structure Analyses. *J. Cryst. Soc. Jpn.* **2009**, *51*, 218.

[14] O. V. Dolomanov, L. J. Bourhis, R. J. Gildea, J. A. K. Howard, Puschmann, H. *J. Appl. Crystallogr.*, **2009**, *42*, 339.
